# Ethnicity-Stratified Normative Retinal Vascular Features from the UK Biobank Using Deep Learning

**DOI:** 10.1016/j.xops.2026.101221

**Published:** 2026-05-08

**Authors:** Ranjit J. Injety, Callum Hunt, Ha-Jun Yoon, Martin D. Tobin, Chiara Batini, Thompson G. Robinson, Kamlesh Khunti, Pearse A. Keane, Robert C. Free, Jatinder S. Minhas, Mervyn G. Thomas

**Affiliations:** 1 The University of Leicester Ulverscroft Eye Unit, School of Psychology and Vision Sciences, University of Leicester, Leicester, UK; 2 University Hospitals of Leicester NHS Trust, Leicester, UK; 3 Division of Public Health and Epidemiology, School of Medical Sciences, University of Leicester, Leicester, UK; 4 Cerebral Haemodynamics in Ageing and Stroke Medicine (CHiASM), Department of Cardiovascular Sciences, University of Leicester, Leicester, UK; 5 NIHR Leicester Biomedical Research Centre, Leicester British Heart Foundation Centre of Research Excellence, Glenfield Hospital, Leicester, UK; 6 Diabetes Research Centre, Leicester General Hospital, University of Leicester, Leicester, UK; 7 NIHR Biomedical Research Centre at Moorfields Eye Hospital NHS Foundation Trust, London, UK; 8 Institute of Ophthalmology, University College London, London, UK; 9 School of Computing and Mathematical Sciences, College of Science and Engineering, University of Leicester, Leicester, UK

**Keywords:** Retinal vascular biomarkers, AutoMorph, Ethnicity, Normative population data, UK biobank

## Abstract

**Purpose:**

Retinal vascular features provide noninvasive biomarkers of systemic vascular health, and deep learning tools such as AutoMorph now enable their large-scale quantification. However, researchers still lack normative data across diverse ethnic populations.

**Design:**

A prospective cohort study.

**Participants:**

We analyzed 6843 UK Biobank participants who reported no disease or medical condition according to the World Health Organization's International Classification of Diseases and Related Health Problems.

**Methods:**

We analyzed retinal images from the UK Biobank using the AutoMorph deep learning pipeline, which extracted retinal morphometric features such as vessel caliber, fractal dimension, vessel density, and tortuosity. We then used these data to define ethnicity-stratified normative ranges for retinal morphometric features in healthy UK Biobank participants. We examined associations with demographic covariates (age, sex, and ethnicity) using multivariate regression.

**Main Outcome Measures:**

We determined normal ranges for retinal morphometric features in the healthy UK Biobank population and assessed associations with age, sex, and ethnicity.

**Results:**

The cohort (mean age 53.5 ± 7.9 years; 50.1% male) was predominantly White participants (91.1%). Retinal vascular complexity declined with age, reflected by lower fractal dimension, vessel density, and tortuosity metrics. Zone-specific analyses confirmed age-related reductions in the central retinal artery equivalent and central retinal vein equivalent. Vessel density (0.050 ± 0.005) and fractal dimension (1.052 ± 0.09) were higher among Chinese participants as compared to White participants (*P* < 0.0001 for both). Across all metrics, ethnicity, followed by sex, exerted the strongest influence on vascular morphometrics.

**Conclusions:**

We established the first ethnicity-stratified normative dataset of retinal vascular features derived from the UK Biobank using deep learning. These data provide a reference framework for oculomic biomarkers in multi-ethnic populations and could support precision-medicine approaches to systemic disease risk assessment.

**Financial Disclosure(s):**

Proprietary or commercial disclosure may be found in the Footnotes and Disclosures at the end of this article.

The retina provides a unique, noninvasive window into systemic vascular health.^1^^,^^2^ Artificial intelligence–based retinal analysis predicts cardiovascular disease with performance comparable to traditional biomarkers.^3^^,^^4^ Clinical implementation requires robust normative data to generate individualized metrics such as the retinal age gap, which associates each year of deviation from expected values with a 2% increase in mortality risk and a 3% increase in cardiovascular disease risk.^5^ By identifying disease-specific biomarkers through artificial intelligence algorithms and defining their normative ranges, clinicians can identify high-risk patients and detect systemic diseases earlier.^6^^,^^7^ In this study, we address this need by establishing ethnicity-stratified normative ranges for retinal vascular features in 6843 UK Biobank participants.

AutoMorph, a publicly available, validated, and fully automated deep learning pipeline, analyses and quantifies retinal vascular morphology from color fundus photographs.^8^ AutoMorph integrates modules for retinal image preprocessing, image-quality grading, anatomical segmentation, and feature measurement. The preprocessing and quality-grading modules standardize image dimensions to a geometric square and ensure adequate image quality. A deep learning network then segments binary vessels (arteries and veins) using an information-fusion framework. AutoMorph subsequently measures vascular features, including vessel tortuosity, fractal dimension, vessel density, and vessel caliber.^8^

In this study, we aim to define ethnicity-specific normative ranges for retinal morphometric features (RMFs) derived from AutoMorph's analysis of UK Biobank data. We also assess how demographic covariates influence these vascular features.

## Methods

### UK Biobank Data

The UK Biobank comprises a large population-based cohort of over half a million participants recruited between January 2006 and October 2010 from 22 assessment centers across the UK. The North West Multi-centre Research Ethics Committee approved the study (REC reference: 21/NW/0157), and all participants provided written informed consent in accordance with the principles of the Declaration of Helsinki. At recruitment, participants were 40 to 69 years old, 54% were female, and 94.6% self-identified as White ethnicity.^9^

Researchers collected demographic, medical, and lifestyle data through questionnaires, physical measurements, and linkage to electronic health records. They coded diagnoses using the World Health Organization's International Classification of Diseases and Related Health Problems.^10^ Technicians acquired retinal images using a 45° single-field color fundus camera centered on the macula and optic disc (Topcon 3D OCT-1000 Mark II, Topcon Corp), imaging the right eye first.^11^^,^^12^ The full study protocol and test descriptions are available online (https://biobank.ndph.ox.ac.uk/ukb/index.cgi).

We extracted anthropometric data (age, sex, self-reported ethnicity, and body mass index [BMI]) (definitions in Table S1, available at www.ophthalmologyscience.org), medical diagnoses (International Classification of Diseases codes), and color fundus photographs from the UK Biobank. We selected the first-ever imaging dataset (instance 0, array 0) of color fundus photographs (n = 175 709). We coded self-reported ethnicity into 5 categories: White, Black, Asian, Chinese, and Other (Table S1).

### Retinal Image Processing by AutoMorph

We processed the color fundus images of the UK Biobank participants (n = 67 279) using the validated AutoMorph deep learning pipeline. The EfficientNet-B4 model graded image quality. AutoMorph's adversarial segmentation network used an information-fusion architecture to segment arteries and veins with a coarse-to-fine approach.^8^

AutoMorph automatically quantified RMFs (definitions in Table S2, available at www.ophthalmologyscience.org), including:

– Optic nerve head parameters: optic disc height, cup height and width, and vertical and horizontal cup-to-disc ratio (CDR).
– Vessel caliber: central retinal artery equivalent, central retinal vein equivalent (CRVE), and the arteriolar–venular ratio.
– Vessel tortuosity: distance tortuosity, squared-curvature tortuosity, and tortuosity density.
– Fractal dimension: Minkowski–Bouligand dimension (vascular branching complexity).
– Vessel density: proportion of retinal area occupied by vessels.

**Figure 1:**
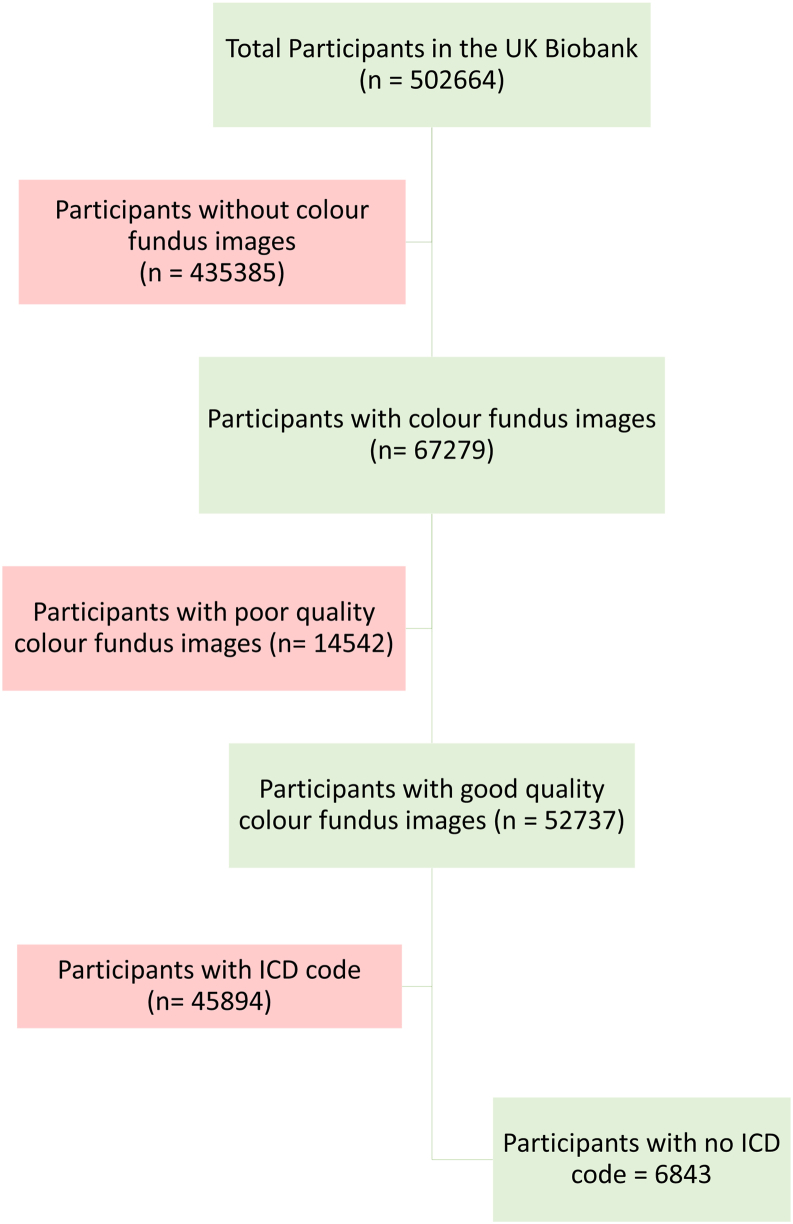
Study flowchart showing the selection of the healthy participants. The normative cohort are the participants with no ICD code. ICD = International Classification of Diseases.

We obtained all measurements within standardized peripapillary regions: zone B (0.5–1 disc diameter from the margin) and zone C (0.5–2 disc diameters).^8^ The measurements for central retinal artery equivalent, CRVE, and average width are in pixels.^8^ The measures were then corrected for ocular magnification and refractive status using Bengtsson's formula.^13^ After segmentation, there were 52 737 participants with good quality color fundus images, which were then filtered for any medical diagnosis. We defined participants with no diagnosed disease or no International Classification of Diseases code as the normative cohort (n = 6843) (see Fig 1).

### Statistical Analysis

We summarized continuous variables as means and standard deviations and categorical variables as counts and percentages. We stratified descriptive statistics for RMFs by age group, sex, BMI, and ethnicity. We analyzed differences in RMFs across ethnicities using analysis of variance. We used multivariable linear regression models to examine associations between RMFs and demographic variables, including ethnicity (White, Black, Asian, Chinese, Mixed, and Other), age group (40–49, 50–59, 60–69, and >70), and sex. We did a subgroup analysis wherein we stratified the RMFs by BMI.

We considered a 2-sided *P* < 0.05 statistically significant and applied a false discovery rate (FDR) correction to adjust for multiple comparisons.

### Ethical Approval

We accessed and analyzed UK Biobank phenotypic data under application 85 881, approved by the North West Multi-centre Research Ethics Committee (REC reference 11/NW/03 820). All participants provided written informed consent.

## Results

### Participant Characteristics

**Figure 2:**
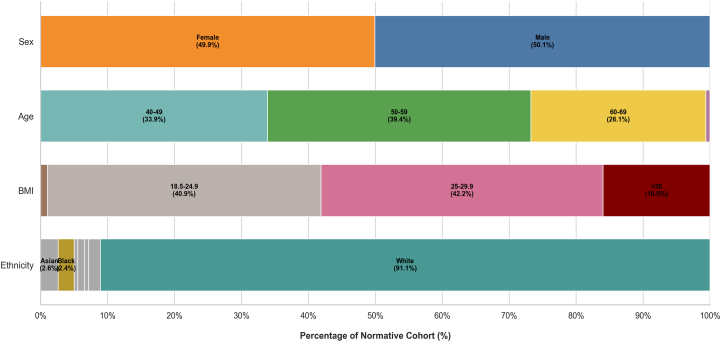
Demographic distribution of the normative cohort. Age group includes 40–49 (2319, 33.9%), 50–59 (2693, 39.4%), 60–69 (1787, 26.1%), and >70 (44, 0.6%). Ethnicity includes White (6233, 91.1%), Asian (179, 2.6%), Black (164, 2.4%), Others (199, 1.7%), Mixed (71, 1%), and Chinese (35, 0.5%). BMI = body mass index.

A total of 6843 participants met the inclusion criteria for the normative cohort. Their mean age was 53.5 ± 7.91 years, with most individuals aged 50 to 59 years (39.4%); only 0.6% were aged ≥70 years. The cohort showed a balanced sex distribution (49.9% male; 50.1% female) and was predominantly White participants (91.1%), with smaller proportions identifying as Asian participants (2.6%), Black participants (2.4%), or other ethnic groups (<2%) (Fig 2).

A 1-way analysis of variance demonstrated significant differences in age across ethnicities, with Black and Asian participants generally younger than White participants (mean age: 48 vs. 54 years; *P* < 0.0001). Among 6815 individuals with available data, the mean BMI was 26.2 ± 4.4 kg/m^2^, indicating a trend toward overweight (43% overweight and 16% obese). However, BMI had no significant impact on the RMFs (see Table S3, available at www.ophthalmologyscience.org).

### Optic Nerve Head Morphology

**Figure 3:**
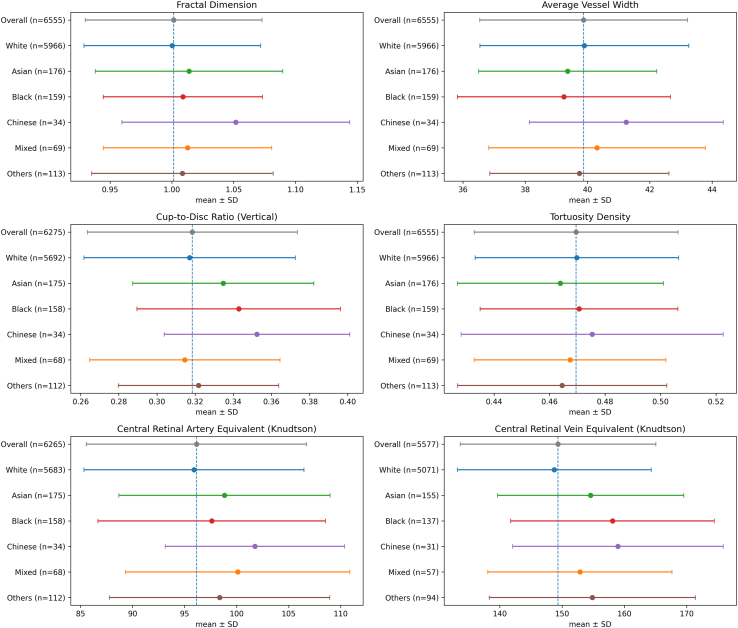
Forest plot showing the differences in the normative ranges of select retinal morphometric features across the ethnicities. SD = standard deviation.

Normative optic disc height and width measured 1065.03 ± 158.97 and 1013.05 ± 156.99, respectively. Optic cup dimensions averaged 506.59 ± 124.84 in height and 489.33 ± 128.32 in width, corresponding to mean vertical and horizontal CDRs of 0.319 ± 0.05 and 0.324 ± 0.05. Significant ethnic variations were observed across all morphology parameters (*P* < 0.001). Chinese and Black participants exhibited the largest disc sizes, whereas White participants exhibited the smallest (disc height: Chinese = 1208.52 ± 137.25; Black = 1183.05 ± 134.05; vs. White = 1056.25 ± 159.06; *P* < 0.0001). Similarly, the Chinese (0.352 ± 0.04), Black (0.343 ± 0.053), and Asian (0.335 ± 0.04) groups all showed significantly higher vertical CDRs compared to White participants (0.317 ± 0.05; *P* < 0.0001), reflecting distinct structural differences in the optic nerve head across ethnicities (Table S4, available at www.ophthalmologyscience.org, Fig 3).

### Retinal Vascular Complexity and Caliber

Across the cohort, the mean fractal dimension was 1.001 ± 0.07, vessel density was 0.047 ± 0.007, and average vessel width was 39.87 ± 3.33 pixels. Subanalysis of vessel types revealed that arteries were narrower (39.42 ± 3.56 pixels) and exhibited lower branching complexity (fractal dimension 0.868 ± 0.06) than veins (43.30 ± 4.31 pixels; fractal dimension 0.885 ± 0.06). Zone-specific analysis showed that both arterial and venous calibers were higher in zone C than in zone B; for example, CRVE was 149.36 ± 15.71 pixels in zone C compared to 120.91 ± 26.23 pixels in zone B (Table S4, Fig 3). Ethnic differences were pronounced in vascular calibers. Central retinal vein equivalent in zone C was largest in Chinese (158.97 ± 16.88 pixels) and Black (158.10 ± 16.33 pixels) participants, significantly exceeding the values for White participants (148.75 ± 15.55 pixels; *P* < 0.0001). Furthermore, Chinese participants displayed the highest vessel density (0.050 ± 0.005) and fractal dimension (1.052 ± 0.09), suggesting a more complex retinal microvascular network in this group compared to White participants (both *P* < 0.0001) (see Fig 3).

### Tortuosity Metrics

The cohort's distance tortuosity averaged 2.288 ± 0.73, squared-curvature tortuosity averaged 16.15 ± 14.22, and tortuosity density averaged 0.470 ± 0.73. Significant ethnic variations were observed for overall distance tortuosity (*P* < 0.0001) and squared-curvature tortuosity (*P* < 0.0001). Chinese and White participants exhibited the highest levels of distance tortuosity (2.311 ± 0.71 and 2.305 ± 0.73, respectively), while participants from the mixed ethnic group exhibited the lowest (2.063 ± 0.60). Chinese (0.475 ± 0.036) and Black (0.471 ± 0.047) participants had higher tortuosity density than the other participants (Table S4, Fig 3). Similar to the caliber results, arterial tortuosity showed significant ethnic variation (artery distance tortuosity, *P* = 0.0001), whereas venous tortuosity metrics, such as vein distance tortuosity (2.328 ± 1.06; *P* = 0.374), did not demonstrate statistically significant differences across the groups (Table S4).

### Multivariable Associations

**Figure 4:**
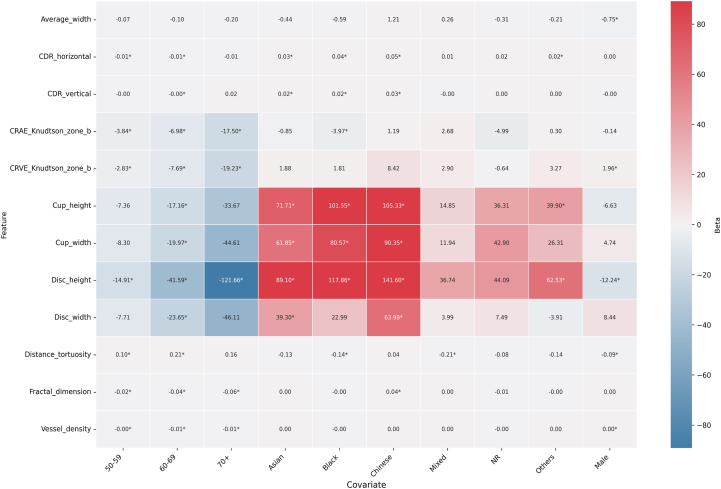
Association between selected retinal morphometric features, age, ethnicity, and sex (FDR corrected). The reference for the covariates used is age 40–49, White ethnicity, and females; FDR *P* < 0.05 are marked with an asterisk. CDR = cup-to-disc ratio; CRAE = central retinal artery equivalent; CRVE = central retinal vein equivalent; FDR = false discovery rate.

After adjusting for ethnicity and sex, age was the strongest determinant of microvascular architecture. Participants aged ≥70 years showed a significant reduction in global fractal dimension (β = –0.063, 95% confidence interval [CI] –0.086 to –0.041; FDR *P* < 0.0001), indicating loss of branching complexity. Individuals aged 60–69 exhibited one of the largest effects in the dataset for vessel density (β = –0.00644, 95% CI –0.00677 to –0.00610; FDR *P* < 0.0001) (see Table S5, available at www.ophthalmologyscience.org, Fig 4). Tortuosity metrics followed similar trends: tortuosity density was lower in the 60–69 group versus 40–49 group (β = –0.00435, 95% CI –0.00671 to –0.00198; FDR *P* = 0.00139), while curvature-based tortuosity frequently increased with age (see Table S5). In contrast, age showed comparatively weak and often nonsignificant FDR-adjusted associations with optic nerve head morphology (disc height/width and cup height/width).

Ethnicity displayed the second strongest associations, especially for optic nerve head morphology and vessel caliber. Disc height was substantially greater in non-White groups: Chinese (β = +141.6, 95% CI 89.1–194.1; FDR *P* < 0.0001), Black (β = +117.9, 95% CI 89.7–146.2; FDR *P* < 0.0001), and Asian (β = +89.2, 95% CI 65.5–113.0; FDR *P* < 0.0001) (see Table S5, Fig 4). Chinese participants also had larger cups (height: β = +105.2, 95% CI 63.8–146.9; FDR *P* < 0.0001; width: β = +90.3, 95% CI 47.5–133.1; FDR *P* = 0.00018) and higher CDRs (vertical: β = +0.034, 95% CI 0.016–0.053; FDR *P* = 0.0012; horizontal: β = +0.050, 95% CI 0.031–0.070; FDR *P* < 0.0001). Venous caliber in zone C was wider in Chinese (β = +3.03, 95% CI 0.924–5.14; FDR *P* = 0.0163); Asian participants had modest density increases in zone C (β = +0.000658, 95% CI 0.000269–0.00105; FDR *P* = 0.00361) (see Table S5).

Sex effects were selective but consistent. Males had narrower average vessels (β = –0.747, 95% CI –0.926 to –0.569; FDR *P* < 0.0001) and narrower arterioles (β = –0.841, 95% CI –1.04 to –0.64; FDR *P* < 0.0001), yet higher CRVE in zone C (β = +2.67, 95% CI 1.86–3.48; FDR *P* < 0.0001) and lower distance tortuosity (β = –0.0966, 95% CI –0.132 to –0.0607; FDR *P* < 0.0001). Optic nerve head sex differences were small and largely nonsignificant after FDR correction (see Table S5, Fig 4).

In summary, ethnicity and sex exert a strong influence on the retinal vascular morphometric measures. Ethnic differences were most pronounced for vessel density and caliber, with Asian, Black, and Other groups generally showing higher vessel density and larger calibers, while male sex was associated with narrower vessels and less tortuous vasculature. Chinese and Black participants tended to exhibit extremes of optic nerve head and vascular measures among the retinal features, with consistently larger disc and cup dimensions and higher vascular density compared with White participants. These normative patterns provide a foundation for future disease-specific comparisons (see Table S4).

## Discussion

This study provides the first ethnicity-stratified normative dataset of retinal vascular and optic nerve head parameters derived from the UK Biobank using the AutoMorph deep learning pipeline. The open-sourced AutoMorph pipeline was preferred because it matched state-of-the-art performance in image grading (F1 score of 0.86), similar F1 scores (0.73 F1 on AV-WIDE, 0.78 F1 on DR-HAGIS) as compared to strong deep learning models and a strong F1 score of 0.94 for optic disc segmentation, which is as good as other segmentation methods (F1 scores between 0.90 and 0.95), thus it is the most widely adopted pipeline in oculomics. It also provides computational transparency, thus allowing for clear traceability of the measures.^8^ By analyzing over 6800 healthy participants, we defined normative ranges for key morphometric features and demonstrated systematic variation by age, sex, and ethnicity. Yusufu et al^14^ established comprehensive normative ranges for 114 retinal measurements in 10 151 UK Biobank participants; however, they did not stratify their findings by ethnicity and included only self-identified White individuals. Given growing evidence that retinal parameters vary significantly across ethnic groups,^15^^,^^16^ our study addresses this gap by providing the first UK Biobank norms stratified by ethnicity. These findings establish a foundation for applying retinal vascular biomarkers in multi-ethnic clinical and population research.

We found that Chinese and Black participants had significantly larger optic disc and cup dimensions as compared to White participants, even after adjusting for age and sex. This observation aligns with previous US studies reporting larger optic disc areas and higher cup-to-disc ratios in individuals of Black descent, which may influence glaucoma risk and diagnostic thresholds.^17^^,^^18^ The smaller disc and cup measurements observed in White participants reinforce the importance of accounting for structural differences when assessing glaucoma and its progression.^19^ Age-related reductions in disc height and cup size reflected neuroretinal rim thinning. Males and females showed similar optic nerve head morphology, although males displayed slightly smaller disc and cup dimensions.^20^

Retinal vascular caliber also varied significantly by ethnicity. Chinese and Black participants exhibited larger arterial diameters, particularly in zone C, as compared to White participants. These findings corroborate prior reports of racial differences in retinal vessel dimensions.^21^^,^^22^ Genetic, developmental, and environmental factors likely drive these patterns. Although genome-wide association studies have identified loci associated with vessel caliber,^23^^,^^24^, self-reported ethnicity reflects a broader interplay of ancestry, environment, socioeconomic influences, and health care access. The persistence of ethnic differences after adjusting for cardiovascular risk factors suggests that additional determinants—including genetic variation and developmental influences—contribute to interethnic variability. Nonetheless, substantial overlap and heterogeneity within ethnic groups emphasize the need for individual-level rather than group-level risk assessment. Central retinal artery equivalent and CRVE both declined with age, indicating progressive arteriolar and venular narrowing. Such vascular attenuation may reflect microvascular aging and has been associated with increased cardiovascular risk.^25^, ^26^, ^27^ Males exhibited narrower vessel calibers and narrow arterioles, but wider CRVE (zone C) when compared to females, consistent with known sex-specific cardiovascular risk profiles.^28^^,^^29^

Fractal dimension and vessel density—indicators of vascular branching and perfusion—were the highest in Asian, Chinese, and Black participants and the lowest in White participants. Both metrics declined markedly with age, especially in those about 70 years, suggesting age-related reductions in vascular complexity and perfusion capacity. These changes may contribute to retinal and neurodegenerative disorders associated with aging.^30^, ^31^, ^32^ Males showed slightly higher fractal dimensions and narrower vessels, which may reflect sex-specific vascular remodeling or hormonal influences.^29^^,^^33^^,^^34^

We observed significantly higher tortuosity values in White and Chinese participants than in other ethnic groups. Increased tortuosity has been linked to vascular stress and systemic diseases such as hypertension and diabetes.^35^, ^36^, ^37^ Tortuosity metrics increased with age, although not significantly, consistent with previous work showing age-related increases in vessel curvature.^37^^,^^38^ Male sex consistently associated with lower tortuosity, suggesting relative vascular resilience or structural differences between sexes.^39^ These findings underscore the importance of contextualizing tortuosity when using it as a biomarker of systemic vascular health.

Age exerted the strongest influence on vascular morphometrics. Aging reduced vascular complexity, narrowed arterioles and venules, decreased fractal dimension, and increased tortuosity. Capillary function showed a quadratic decline after age 50. Each 1-year increase in retinal age gap has been linked to a 2% increase in all-cause mortality (hazard ratio = 1.02) and a 3% increase in cardiovascular disease risk (hazard ratio = 1.03).^40^

Ethnicity contributed to marked heterogeneity in optic nerve head geometry and vascular complexity, consistent with published reports of ethnic variation in retinal morphology.^31^^,^^41^ Sex-related differences in vessel caliber and fractal metrics also aligned with evidence on hormonal and hemodynamic influences on retinal vasculature.^28^^,^^33^^,^^42^

Our study offers several strengths. First, it provides the first ethnicity-stratified normative dataset from the UK Biobank, offering a valuable reference for ethnicity-specific analyses of retinal vascular disease. Second, the use of high-quality fundus imaging enhances the statistical reliability of the normative estimates across ethnic groups. Third, the inclusion of demographic and systemic covariates enables clinically relevant interpretation of retinal vascular variation.

However, our study also has limitations. We derived normative ranges solely from UK Biobank data, which included relatively small sample sizes for non-White groups. Additionally, we classified participants as healthy based on the absence of recorded diagnoses, which may overlook undiagnosed or subclinical disease. The age range of 40–70 years limits generalizability to younger and older populations. Future population-based studies are needed to evaluate prospective clinical applicability and to capture additional context that may influence retinal metrics. Finally, retinal vascular parameters vary across algorithms, including AutoMorph and other pipelines, which may limit comparability across studies. We acknowledge that although AutoMorph may not be the most superior pipeline, it is as reliable as other models, in addition to having computational transparency. Future work should assess concordance across analytic methods and incorporate longitudinal, genetic, and systemic data to improve early detection and risk prediction.

## Conclusion

This study establishes ethnicity-stratified normative reference ranges for retinal vascular and optic nerve head features in healthy UK Biobank participants using a validated deep learning approach. These benchmarks show how age, sex, and ethnicity shape retinal microvascular architecture and provide essential context for interpreting retinal biomarkers in clinical and research settings.

The ethnic variation we observed in optic disc and vascular morphology, along with age-related reductions in vascular complexity and increases in tortuosity, underscores the importance of incorporating demographic context into oculomic analyses. Defining such normative data represents a critical step toward translating retinal imaging into precision-medicine tools for predicting systemic and neurovascular disease risk.

Future work should integrate longitudinal data and combine genetic, metabolic, and systemic health information to refine risk modeling and clarify the mechanisms linking retinal and systemic vascular health.
