# Supplementary material for: Ethnicity-Stratified Normative Retinal Vascular Features from the UK Biobank Using Deep Learning
Source: Ophthalmol Sci. 2026 May 8;6(7):101221. doi: 10.1016/j.xops.2026.101221 (PMC13260094; doi:10.1016/j.xops.2026.101221)
Supplement: Table S1 [file mmc1.pdf]

Table S1. Definitions of variables used in the current study

Variable	Definition and collection	Link
Age	Age when imaging was done	https://biobank.ndph.ox.ac.uk/showcase/field.cgi?id=21003
Sex	Sex of participant. Acquired from central registry at recruitment, but in some cases updated by the participant. Hence this field may contain a mixture of the sex the NHS had recorded for the participant and self-reported sex.	http://biobank.ndph.ox.ac.uk/ukb/field.cgi?id=31
Ethnicity	This is an amalgam of sequential branching questions asked during the initial Assessment Centre visit as part of the touchscreen questionnaire. The question was dropped from the touchscreen protocol on 24/10/2016.<br>The ethnic categories included:<br>White includes British, Irish, and any other white background<br>Black includes African, Black or Black British, Caribbean and any other Black background<br>Asian includes Indian, Pakistani, Bangladeshi, and any other Asian background<br>Mixed includes White and Black Caribbean, White and Black African, White and Asian, and any other mixed background<br>Others includes other ethnic groups	http://biobank.ndph.ox.ac.uk/ukb/field.cgi?id=21000
Body mass index (BMI)	Defined as weight in kilogrammes divided by height in metres squared. BMI value here is constructed from height and weight measured during the initial Assessment Centre visit. Value is not present if either of these readings were omitted.	http://biobank.ndph.ox.ac.uk/ukb/field.cgi?id=21001
