# Supplementary material for: Ethnicity-Stratified Normative Retinal Vascular Features from the UK Biobank Using Deep Learning
Source: Ophthalmol Sci. 2026 May 8;6(7):101221. doi: 10.1016/j.xops.2026.101221 (PMC13260094; doi:10.1016/j.xops.2026.101221)
Supplement: Table S2 [file mmc2.pdf]

## Table S2. Definitions of measure types

| Category | Measure Type | Definition |
|---|---|---|
| Disc/cup | Disc size | the dimensions (horizontal and vertical) and area of the optic nerve head, the point where nerve fibers exit the eye, typically an oval shape. |
| | Cup size | the dimension of the central depression in the optic nerve head (disc) at the back of the eye |
| | Cup-to-disc ratio | the ratio of the diameter of the central, optic cup (the depression in the nerve head) to the entire optic disc (the whole nerve head where the optic nerve enters the eye) |
| Tortuosity | Distance Tortuosity | the ratio of the actual path length of the vessel to the straight-line distance between its endpoints |
| | Squared curvature tortuosity | the intensity of vessel bends at each point, squaring these values to emphasize sharpness, and then summing them along the entire segment. |
| | Tortuosity density | the sum of individual vessel tortuosity normalized by the area or length of the vessels within that area. |
| Calibre | Central Retinal Arteriolar Equivalent (CRAE) | a quantitative measure used in ophthalmology to estimate the average diameter of the retinal arterioles in the eye. CRAE is derived from the calibres of the retinal arterioles using the Parr-Hubbard or the Knudtson formula. Measured in pixels. |
| | Central Retinal Venular Equivalent (CRVE) | a quantitative measure used in ophthalmology to estimate the average diameter of the retinal venules in the eye. This metric is derived from the measurements of the calibres (widths) of individual retinal veins using the Parr-Hubbard or the Knudtson formula. Measured in pixels. |
| | Arteriole-to-Venule Ratio (AVR) | a quantitative metric used in ophthalmology to assess the relative diameters of retinal arterioles (small arteries) and venules (small veins) |
| Other | Fractal Dimension | a quantitative measure used to describe the complex, branching patterns observed in the retinal vasculature. The fractal dimension value (Minkowski–Bouligand dimension) provides a measurement of vessel complexity. |
| | Vessel density | the percentage area occupied by the large vessels and microvasculature in a specific area. |
| | Average width | measure of the width of the vessels across the zones in pixels |
