# Supplementary material for: Ethnicity-Stratified Normative Retinal Vascular Features from the UK Biobank Using Deep Learning
Source: Ophthalmol Sci. 2026 May 8;6(7):101221. doi: 10.1016/j.xops.2026.101221 (PMC13260094; doi:10.1016/j.xops.2026.101221)
Supplement: Table S3 [file mmc3.pdf]

Table S3. Impact of BMI on the Retinal Morphometric Features by Ethnicity

Feature	Overall p-value	White p-value	Black p-value	Asian p-value	Chinese p-value	Mixed p-value	Others p-value	Overall FDR p-value	White FDR p-value	Black FDR p-value	Asian FDR p-value	Chinese FDR p-value	Mixed FDR p-value	Others FDR p-value
**Disc height**	0.0895	0.0107	0.8956	0.6645	0.1566	0.1348	0.9582	0.3091	0.0895	0.9634	0.8168	0.4114	0.3794	0.9796
**Disc width**	0.5434	0.2899	0.7377	0.3983	0.0117	0.1248	0.1946	0.7422	0.5433	0.8613	0.6279	0.0930	0.3723	0.4420
**Cup height**	0.1644	0.0249	0.5223	0.3128	0.2929	0.2118	0.2785	0.4173	0.1512	0.7232	0.5629	0.5442	0.4601	0.5357
**Cup width**	0.5680	0.1950	0.4370	0.4514	0.0364	0.3400	0.2406	0.7550	0.4420	0.6536	0.6644	0.1800	0.5927	0.4950
**CDR vertical**	0.1362	0.0678	0.1535	0.0562	0.0271	0.4842	0.1417	0.3814	0.2560	0.4096	0.2310	0.1617	0.6919	0.3859
**CDR horizontal**	0.1546	0.0455	0.1578	0.2445	0.0135	0.8535	0.6365	0.4101	0.2049	0.4114	0.5003	0.0976	0.9346	0.8040
**Fractal dimension**	0.0008	0.0013	0.0498	0.0814	0.0135	0.6565	0.9668	0.0125	0.0171	0.2177	0.2910	0.0976	0.8090	0.9844
**Vessel density**	0.0000	0.0000	0.2679	0.2490	0.6911	0.3727	0.7855	0.0001	0.0002	0.5258	0.5060	0.8352	0.6079	0.8916
**Average width**	0.0000	0.0000	0.5134	0.0716	0.4846	0.2014	0.0128	0.0000	0.0003	0.7167	0.2636	0.6919	0.4484	0.0975
**Distance tortuosity**	0.1095	0.1248	0.8893	0.6539	0.1093	0.9419	0.9916	0.3516	0.3723	0.9598	0.8090	0.3516	0.9749	0.9952
**Squared curvature tortuosity**	0.1849	0.1849	0.4500	0.4724	0.2697	0.5155	0.9503	0.4355	0.4355	0.6644	0.6802	0.5258	0.7177	0.9772
**Tortuosity density**	0.0136	0.0335	0.1799	0.1135	0.0184	0.9520	0.1339	0.0976	0.1715	0.4355	0.3596	0.1203	0.9772	0.3794
**Artery Fractal dimension**	0.0000	0.0000	0.0285	0.0714	0.0234	0.5612	0.9093	0.0002	0.0006	0.1618	0.2636	0.1437	0.7508	0.9709
**Artery Vessel density**	0.0000	0.0000	0.2515	0.1178	0.2999	0.3578	0.9692	0.0000	0.0000	0.5071	0.3659	0.5496	0.6045	0.9848
**Artery Average width**	0.0000	0.0000	0.6487	0.0593	0.6425	0.3482	0.0501	0.0000	0.0000	0.8090	0.2391	0.8055	0.6010	0.2177
**Artery Distance tortuosity**	0.3706	0.7459	0.1128	0.5838	0.0019	0.4220	0.0333	0.6064	0.8622	0.3596	0.7623	0.0226	0.6445	0.1715
**Artery Squared curvature tortuosity**	0.7706	0.4108	0.2900	0.6390	0.0392	0.3646	0.0999	0.8792	0.6371	0.5433	0.8040	0.1899	0.6064	0.3355
**Artery Tortuosity density**	0.0134	0.0646	0.5716	0.0304	0.1386	0.5975	0.1095	0.0976	0.2523	0.7550	0.1684	0.3853	0.7721	0.3516
**Vein Fractal dimension**	0.0166	0.0165	0.0552	0.1410	0.0222	0.8378	0.7647	0.1143	0.1143	0.2310	0.3859	0.1396	0.9220	0.8759
**Vein Vessel density**	0.2319	0.3626	0.5488	0.8042	0.5862	0.9394	0.2452	0.4830	0.6064	0.7440	0.9048	0.7635	0.9749	0.5003
**Vein Average width**	0.3344	0.1758	0.8765	0.1944	0.2599	0.0276	0.1235	0.5851	0.4300	0.9542	0.4420	0.5199	0.1617	0.3723
**Vein Distance tortuosity**	0.0748	0.0735	0.2684	0.5922	0.8886	0.4314	0.8249	0.2711	0.2683	0.5258	0.7672	0.9598	0.6510	0.9137

Feature	Overall p-value	White p-value	Black p-value	Asian p-value	Chinese p-value	Mixed p-value	Others p-value	Overall FDR p-value	White FDR p-value	Black FDR p-value	Asian FDR p-value	Chinese FDR p-value	Mixed FDR p-value	Others FDR p-value
**Vein Squared curvature tortuosity**	0.0569	0.0508	0.5393	0.9813	0.9002	0.1238	0.6311	0.2314	0.2190	0.7401	0.9911	0.9647	0.3723	0.8032
**Vein Tortuosity density**	0.0016	0.0038	0.0908	0.2216	0.0104	0.5100	0.1583	0.0210	0.0396	0.3113	0.4704	0.0895	0.7140	0.4114
**Fractal dimension zone b**	0.0205	0.0229	0.1826	0.2671	0.0564	0.7024	0.8525	0.1308	0.1428	0.4355	0.5258	0.2310	0.8429	0.9346
**Vessel density zone b**	0.0180	0.0086	0.9918	0.7710	0.4278	0.4150	0.9847	0.1203	0.0785	0.9952	0.8792	0.6494	0.6377	0.9926
**Average width zone b**	0.0010	0.0044	0.6383	0.1608	0.0686	0.8965	0.0932	0.0142	0.0437	0.8040	0.4134	0.2560	0.9634	0.3173
**Distance tortuosity zone b**	0.8828	0.7583	0.1980	0.0786	0.0627	0.9219	0.6533	0.9585	0.8706	0.4452	0.2829	0.2468	0.9715	0.8090
**Squared curvature tortuosity zone b**	0.6268	0.7188	0.2925	0.0315	0.1532	0.6768	0.3151	0.7998	0.8530	0.5442	0.1706	0.4096	0.8219	0.5629
**Tortuosity density zone b**	0.0017	0.0049	0.1048	0.1569	0.2659	0.0348	0.0682	0.0210	0.0466	0.3476	0.4114	0.5258	0.1753	0.2560
**Artery Fractal dimension zone b**	0.0152	0.0323	0.1391	0.1956	0.0286	0.7478	0.4902	0.1081	0.1707	0.3853	0.4420	0.1618	0.8624	0.6955
**Artery Vessel density zone b**	0.0000	0.0000	0.7258	0.8843	0.7978	0.9420	0.8359	0.0011	0.0008	0.8530	0.9585	0.8996	0.9749	0.9218
**Artery Average width zone b**	0.0000	0.0000	0.6751	0.1874	0.9932	0.3892	0.9233	0.0000	0.0000	0.8219	0.4358	0.9952	0.6276	0.9715
**Artery Distance tortuosity zone b**	0.2022	0.2029	0.2207	0.4589	0.3208	0.3207	0.5506	0.4484	0.4484	0.4704	0.6684	0.5673	0.5673	0.7440
**Artery Squared curvature tortuosity zone b**	0.6066	0.4887	0.0516	0.1923	0.3143	0.9351	0.9339	0.7779	0.6955	0.2203	0.4420	0.5629	0.9738	0.9738
**Artery Tortuosity density zone b**	0.0039	0.0079	0.7236	0.1064	0.0119	0.7403	0.1344	0.0398	0.0742	0.8530	0.3506	0.0930	0.8613	0.3794
**CRAE Hubbard zone b**	0.0005	0.0008	0.3678	0.5266	0.1323	0.8123	0.4264	0.0093	0.0129	0.6064	0.7271	0.3794	0.9093	0.6492
**CRAE Knudtson zone b**	0.0005	0.0008	0.3704	0.5551	0.1338	0.8183	0.4575	0.0093	0.0126	0.6064	0.7480	0.3794	0.9104	0.6684
**Vein Fractal dimension zone b**	0.3256	0.2318	0.2900	0.1987	0.1807	0.5616	0.5449	0.5717	0.4830	0.5433	0.4452	0.4355	0.7508	0.7422
**Vein Vessel density zone b**	0.0682	0.2988	0.6723	0.6154	0.2337	0.1656	0.3158	0.2560	0.5496	0.8219	0.7872	0.4846	0.4173	0.5629
**Vein Average width zone b**	0.0481	0.0120	0.9771	0.3646	0.0877	0.8211	0.2067	0.2146	0.0930	0.9909	0.6064	0.3069	0.9116	0.4509
**Vein Distance tortuosity zone b**	0.8137	0.4438	0.9016	0.4576	0.7517	0.5003	0.5634	0.9093	0.6617	0.9647	0.6684	0.8649	0.7023	0.7512
**Vein Squared curvature tortuosity zone b**	0.6897	0.4828	0.5888	0.3527	0.7209	0.3430	0.6551	0.8352	0.6919	0.7649	0.6038	0.8530	0.5940	0.8090
**Vein Tortuosity density zone b**	0.7416	0.9219	0.1184	0.2944	0.1184	0.0108	0.3107	0.8613	0.9715	0.3659	0.5442	0.3659	0.0895	0.5629
**CRVE Hubbard zone b**	0.1191	0.0599	0.9504	0.0500	0.2040	0.5220	0.9161	0.3659	0.2396	0.9772	0.2177	0.4489	0.7232	0.9715

Feature	Overall p-value	White p-value	Black p-value	Asian p-value	Chinese p-value	Mixed p-value	Others p-value	Overall FDR p-value	White FDR p-value	Black FDR p-value	Asian FDR p-value	Chinese FDR p-value	Mixed FDR p-value	Others FDR p-value
**CRVE Knudtson zone b**	0.0887	0.0448	0.9577	0.0864	0.1752	0.5571	0.9290	0.3081	0.2044	0.9796	0.3045	0.4300	0.7488	0.9734
**AVR Hubbard zone b**	0.4196	0.1603	0.4014	0.1649	0.7094	0.4134	0.1471	0.6427	0.4134	0.6283	0.4173	0.8493	0.6371	0.3986
**AVR Knudtson zone b**	0.0106	0.0096	0.3931	0.1754	0.8328	0.4005	0.1403	0.0895	0.0867	0.6279	0.4300	0.9205	0.6283	0.3859
**Fractal dimension zone c**	0.0035	0.0040	0.1708	0.1270	0.0320	0.5507	0.9650	0.0395	0.0404	0.4262	0.3744	0.1707	0.7440	0.9844
**Vessel density zone c**	0.0117	0.0038	0.6512	0.2861	0.1731	0.3411	0.5992	0.0930	0.0396	0.8090	0.5433	0.4298	0.5927	0.7724
**Average width zone c**	0.0004	0.0033	0.2715	0.3161	0.4332	0.9169	0.0447	0.0069	0.0383	0.5264	0.5629	0.6510	0.9715	0.2044
**Distance tortuosity zone c**	0.0362	0.0299	0.5760	0.7251	0.1151	0.9970	0.9187	0.1800	0.1675	0.7560	0.8530	0.3626	0.9970	0.9715
**Squared curvature tortuosity zone c**	0.2832	0.1818	0.4056	0.7971	0.4340	0.5345	0.7326	0.5406	0.4355	0.6328	0.8996	0.6510	0.7360	0.8586
**Tortuosity density zone c**	0.0012	0.0045	0.4133	0.0975	0.3910	0.7397	0.0450	0.0166	0.0437	0.6371	0.3297	0.6276	0.8613	0.2044
**Artery Fractal dimension zone c**	0.0001	0.0001	0.1891	0.1685	0.0520	0.6710	0.6949	0.0011	0.0026	0.4373	0.4224	0.2204	0.8219	0.8379
**Artery Vessel density zone c**	0.0000	0.0000	0.6376	0.4941	0.2500	0.5703	0.9805	0.0000	0.0000	0.8040	0.6975	0.5060	0.7550	0.9911
**Artery Average width zone c**	0.0000	0.0000	0.2656	0.1021	0.5837	0.5404	0.3605	0.0000	0.0000	0.5258	0.3407	0.7623	0.7401	0.6056
**Artery Distance tortuosity zone c**	0.0037	0.0015	0.7911	0.2204	0.0400	0.7791	0.2535	0.0396	0.0195	0.8960	0.4704	0.1902	0.8864	0.5090
**Artery Squared curvature tortuosity zone c**	0.0415	0.0285	0.3222	0.3960	0.3678	0.2776	0.4454	0.1957	0.1618	0.5678	0.6279	0.6064	0.5357	0.6622
**Artery Tortuosity density zone c**	0.0001	0.0010	0.4719	0.0101	0.3956	0.3114	0.3586	0.0027	0.0144	0.6802	0.0894	0.6279	0.5629	0.6045
**CRAE Hubbard zone c**	0.0000	0.0000	0.6736	0.1243	0.3798	0.9275	0.8175	0.0000	0.0000	0.8219	0.3723	0.6159	0.9734	0.9104
**CRAE Knudtson zone c**	0.0000	0.0000	0.7261	0.1317	0.3986	0.9339	0.9128	0.0000	0.0000	0.8530	0.3794	0.6279	0.9738	0.9715
**Vein Fractal dimension zone c**	0.0654	0.0325	0.1656	0.7111	0.0449	0.1082	0.2795	0.2537	0.1707	0.4173	0.8493	0.2044	0.3516	0.5357
**Vein Vessel density zone c**	0.0824	0.3525	0.2196	0.3558	0.3685	0.1877	0.1322	0.2925	0.6038	0.4704	0.6038	0.6064	0.4358	0.3794
**Vein Average width zone c**	0.1838	0.0662	0.7452	0.8114	0.7166	0.6397	0.0386	0.4355	0.2546	0.8622	0.9093	0.8530	0.8040	0.1887
**Vein Distance tortuosity zone c**	0.2065	0.1332	0.8756	0.4292	0.5735	0.4468	0.9500	0.4509	0.3794	0.9542	0.6496	0.7550	0.6623	0.9772
**Vein Squared curvature tortuosity zone c**	0.4622	0.2246	0.8548	0.2948	0.1536	0.7009	0.4522	0.6713	0.4736	0.9346	0.5442	0.4096	0.8429	0.6644
**Vein Tortuosity density zone c**	0.1825	0.2896	0.3546	0.0204	0.4912	0.2127	0.1270	0.4355	0.5433	0.6038	0.1308	0.6955	0.4601	0.3744

Feature	Overall p-value	White p-value	Black p-value	Asian p-value	Chinese p-value	Mixed p-value	Others p-value	Overall FDR p-value	White FDR p-value	Black FDR p-value	Asian FDR p-value	Chinese FDR p-value	Mixed FDR p-value	Others FDR p-value
**CRVE Hubbard zone c**	0.0007	0.0018	0.3013	0.0276	0.4676	0.3902	0.6516	0.0114	0.0218	0.5502	0.1617	0.6773	0.6276	0.8090
**CRVE Knudtson zone c**	0.0005	0.0017	0.2360	0.0312	0.5738	0.3539	0.6016	0.0094	0.0213	0.4875	0.1706	0.7550	0.6038	0.7735
**AVR Hubbard zone c**	0.0398	0.0183	0.3800	0.4071	0.2221	0.0615	0.2319	0.1902	0.1203	0.6159	0.6332	0.4704	0.2440	0.4830
**AVR Knudtson zone c**	0.0337	0.0174	0.3986	0.4963	0.1858	0.0556	0.2702	0.1715	0.1184	0.6279	0.6987	0.4355	0.2310	0.5258
