# Supplementary material for: Ethnicity-Stratified Normative Retinal Vascular Features from the UK Biobank Using Deep Learning
Source: Ophthalmol Sci. 2026 May 8;6(7):101221. doi: 10.1016/j.xops.2026.101221 (PMC13260094; doi:10.1016/j.xops.2026.101221)
Supplement: Table S4 [file mmc4.pdf]

Table S4. Mean (standard deviation) of the Retinal Morphometric Features by Ethnicity

Feature	Overall	White	Black	Asian	Chinese	Mixed	Others	F_stat	p_value	FDR_p
**Disc height**	1065.030 (158.974)	1056.251 (159.064)	1183.049 (134.059)	1153.378 (124.003)	1208.515 (137.253)	1104.446 (125.399)	1127.390 (117.172)	42.83255	1.52E-43	1.09E-41
**Disc width**	1013.051 (156.993)	1010.486 (159.952)	1040.396 (121.369)	1056.871 (119.717)	1078.212 (149.358)	1018.443 (107.758)	1011.067 (111.514)	5.191046	9.30E-05	0.000279
**Cup height**	506.586 (124.846)	500.057 (123.257)	605.131 (131.349)	574.797 (115.082)	609.973 (117.426)	519.839 (123.369)	543.668 (106.197)	41.29514	5.94E-42	2.14E-40
**Cup width**	489.330 (128.326)	483.796 (127.924)	570.139 (132.001)	551.380 (109.823)	577.763 (140.737)	499.578 (104.796)	514.048 (103.756)	27.5359	1.14E-27	1.43E-26
**CDR vertical**	0.319 (0.055)	0.317 (0.055)	0.343 (0.053)	0.335 (0.047)	0.352 (0.049)	0.315 (0.050)	0.322 (0.042)	12.78255	2.18E-12	1.31E-11
**CDR horizontal**	0.324 (0.059)	0.321 (0.059)	0.367 (0.057)	0.351 (0.049)	0.373 (0.062)	0.328 (0.044)	0.340 (0.047)	34.50175	6.60E-35	1.19E-33
**Fractal dimension**	1.001 (0.072)	1.000 (0.071)	1.009 (0.064)	1.014 (0.076)	1.052 (0.092)	1.013 (0.068)	1.008 (0.073)	5.630361	3.48E-05	0.000119
**Vessel density**	0.047 (0.007)	0.047 (0.007)	0.048 (0.006)	0.049 (0.006)	0.050 (0.005)	0.050 (0.007)	0.050 (0.007)	10.18076	9.88E-10	5.08E-09
**Average width**	39.874 (3.336)	39.898 (3.355)	39.241 (3.420)	39.362 (2.859)	41.240 (3.111)	40.304 (3.476)	39.738 (2.876)	3.442814	0.004144	0.009625
**Distance tortuosity**	2.288 (0.737)	2.305 (0.738)	2.096 (0.670)	2.111 (0.783)	2.311 (0.714)	2.063 (0.602)	2.126 (0.681)	7.20913	9.70E-07	3.88E-06
**Squared curvature tortuosity**	16.147 (14.226)	16.439 (14.549)	12.949 (9.379)	13.356 (10.719)	14.534 (11.863)	11.784 (8.443)	13.231 (9.107)	5.810057	2.33E-05	8.82E-05
**Tortuosity density**	0.470 (0.037)	0.470 (0.037)	0.471 (0.036)	0.464 (0.037)	0.475 (0.047)	0.467 (0.035)	0.465 (0.038)	1.564706	0.166402	0.206568
**Artery Fractal dimension**	0.868 (0.062)	0.867 (0.062)	0.870 (0.055)	0.878 (0.066)	0.913 (0.083)	0.880 (0.058)	0.875 (0.064)	5.604307	3.69E-05	0.000121
**Artery Vessel density**	0.020 (0.003)	0.020 (0.003)	0.020 (0.003)	0.021 (0.003)	0.022 (0.003)	0.022 (0.003)	0.022 (0.003)	13.56845	3.38E-13	2.21E-12
**Artery Average width**	39.417 (3.561)	39.387 (3.570)	39.492 (3.693)	39.495 (3.368)	41.311 (3.434)	40.103 (3.505)	39.800 (3.364)	2.808545	0.015415	0.027747
**Artery Distance tortuosity**	3.671 (2.123)	3.707 (2.133)	3.399 (1.751)	3.011 (1.431)	3.653 (2.060)	3.211 (2.557)	3.426 (2.046)	5.266937	7.85E-05	0.000246
**Artery Squared curvature tortuosity**	47.346 (65.466)	48.123 (65.478)	42.671 (53.257)	31.135 (40.347)	43.668 (62.029)	39.420 (108.636)	43.423 (67.928)	2.808565	0.015414	0.027747
**Artery Tortuosity density**	0.504 (0.047)	0.505 (0.047)	0.498 (0.046)	0.495 (0.046)	0.503 (0.054)	0.498 (0.043)	0.492 (0.043)	4.099981	0.001019	0.002821
**Vein Fractal dimension**	0.885 (0.066)	0.883 (0.066)	0.898 (0.060)	0.900 (0.068)	0.933 (0.079)	0.896 (0.063)	0.895 (0.068)	8.46999	5.31E-08	2.55E-07
**Vein Vessel density**	0.024 (0.004)	0.024 (0.004)	0.026 (0.004)	0.026 (0.004)	0.027 (0.004)	0.025 (0.004)	0.026 (0.005)	27.50501	1.19E-27	1.43E-26
**Vein Average width**	43.299 (4.312)	43.351 (4.352)	42.437 (4.013)	42.592 (3.732)	44.993 (3.926)	43.143 (3.988)	42.709 (3.757)	3.880542	0.001634	0.004201
**Vein Distance tortuosity**	2.328 (1.069)	2.334 (1.073)	2.187 (1.148)	2.303 (0.981)	2.288 (0.977)	2.135 (0.929)	2.339 (1.029)	1.071347	0.374021	0.420773
**Vein Squared curvature tortuosity**	17.863 (22.204)	17.984 (22.363)	15.266 (19.280)	17.853 (24.225)	13.796 (10.588)	13.690 (12.944)	17.813 (19.779)	1.18775	0.312357	0.357071
**Vein Tortuosity density**	0.510 (0.042)	0.510 (0.042)	0.515 (0.044)	0.510 (0.043)	0.527 (0.047)	0.511 (0.037)	0.509 (0.041)	1.648472	0.143544	0.181319
**Fractal dimension zone b**	0.767 (0.062)	0.767 (0.062)	0.770 (0.054)	0.773 (0.062)	0.795 (0.074)	0.769 (0.063)	0.769 (0.060)	1.78476	0.112316	0.15258
**Vessel density zone b**	0.005 (0.001)	0.005 (0.001)	0.005 (0.001)	0.005 (0.001)	0.005 (0.001)	0.005 (0.001)	0.005 (0.001)	3.790177	0.001984	0.004925
**Average width zone b**	49.115 (4.802)	49.098 (4.820)	48.659 (4.453)	49.131 (4.835)	52.165 (4.891)	50.085 (4.370)	49.277 (4.354)	3.625805	0.002815	0.006756
**Distance tortuosity zone b**	1.809 (0.978)	1.806 (0.979)	1.930 (1.095)	1.853 (0.904)	1.744 (0.829)	1.739 (0.891)	1.790 (0.969)	0.669193	0.646826	0.695097
**Squared curvature tortuosity zone b**	6.331 (7.297)	6.286 (7.080)	8.476 (13.437)	6.555 (6.659)	4.863 (4.249)	6.652 (8.576)	5.699 (6.861)	3.275781	0.005885	0.012613
**Tortuosity density zone b**	0.448 (0.055)	0.449 (0.054)	0.453 (0.058)	0.447 (0.058)	0.456 (0.053)	0.447 (0.046)	0.434 (0.061)	1.980112	0.078263	0.117395
**Artery Fractal dimension zone b**	0.645 (0.056)	0.645 (0.056)	0.640 (0.049)	0.647 (0.058)	0.671 (0.070)	0.649 (0.054)	0.645 (0.056)	1.697473	0.131519	0.172171
**Artery Vessel density zone b**	0.002 (0.000)	0.002 (0.000)	0.002 (0.000)	0.002 (0.000)	0.002 (0.000)	0.002 (0.001)	0.002 (0.001)	2.995311	0.010525	0.019942

Feature	Overall	White	Black	Asian	Chinese	Mixed	Others	F_stat	p_value	FDR_p
**Artery Average width zone b**	45.801 (5.384)	45.700 (5.406)	46.499 (5.252)	46.910 (5.085)	48.423 (4.362)	46.485 (5.107)	46.611 (4.574)	4.778139	0.000232	0.000668
**Artery Distance tortuosity zone b**	1.797 (1.459)	1.807 (1.468)	1.755 (1.425)	1.761 (1.385)	1.550 (1.203)	1.781 (1.518)	1.618 (1.293)	0.629941	0.676924	0.706355
**Artery Squared curvature tortuosity zone b**	7.191 (12.915)	7.278 (12.976)	6.842 (11.416)	7.267 (16.328)	3.893 (7.061)	6.362 (10.940)	5.647 (8.890)	0.890195	0.486539	0.538936
**Artery Tortuosity density zone b**	0.497 (0.071)	0.498 (0.071)	0.497 (0.075)	0.495 (0.072)	0.488 (0.063)	0.490 (0.058)	0.479 (0.071)	1.772533	0.114843	0.153124
**CRAE Hubbard zone b**	91.113 (21.560)	91.049 (21.311)	89.025 (26.110)	91.999 (23.945)	94.400 (25.403)	95.946 (19.527)	93.283 (21.317)	1.41891	0.213876	0.256652
**CRAE Knudtson zone b**	80.297 (19.168)	80.251 (18.945)	78.217 (23.195)	81.141 (21.275)	83.038 (22.468)	84.581 (17.649)	82.031 (18.984)	1.433397	0.208689	0.254672
**Vein Fractal dimension zone b**	0.649 (0.056)	0.647 (0.056)	0.662 (0.054)	0.660 (0.055)	0.677 (0.064)	0.651 (0.065)	0.655 (0.057)	5.701002	2.98E-05	0.000107
**Vein Vessel density zone b**	0.002 (0.000)	0.002 (0.000)	0.003 (0.001)	0.003 (0.000)	0.003 (0.001)	0.002 (0.001)	0.003 (0.001)	26.35211	1.94E-26	2.00E-25
**Vein Average width zone b**	53.394 (6.709)	53.399 (6.755)	52.492 (5.671)	53.178 (6.176)	56.335 (6.596)	54.896 (6.269)	53.436 (6.846)	2.593952	0.023762	0.039788
**Vein Distance tortuosity zone b**	1.728 (1.339)	1.711 (1.323)	2.010 (1.626)	2.023 (1.478)	1.797 (1.294)	1.657 (1.469)	1.802 (1.411)	3.417021	0.004377	0.009847
**Vein Squared curvature tortuosity zone b**	6.079 (9.550)	5.987 (9.487)	8.394 (11.873)	7.683 (10.980)	6.238 (8.485)	5.273 (9.449)	5.322 (6.954)	3.186677	0.007085	0.014171
**Vein Tortuosity density zone b**	0.472 (0.072)	0.472 (0.072)	0.472 (0.079)	0.477 (0.072)	0.503 (0.060)	0.459 (0.072)	0.467 (0.076)	1.944523	0.083651	0.122916
**CRVE Hubbard zone b**	113.807 (24.158)	113.433 (24.214)	117.283 (23.348)	117.248 (20.853)	122.356 (24.242)	117.019 (25.773)	117.694 (25.266)	3.270072	0.005956	0.012613
**CRVE Knudtson zone b**	120.913 (26.236)	120.511 (26.283)	124.454 (25.534)	124.552 (22.800)	130.272 (26.887)	124.846 (27.970)	125.216 (27.553)	3.249927	0.006212	0.012779
**AVR Hubbard zone b**	1.365 (4.803)	1.418 (4.721)	1.194 (4.317)	0.433 (5.499)	0.464 (0.182)	1.424 (11.454)	0.749 (1.839)	2.100907	0.062288	0.09542
**AVR Knudtson zone b**	0.818 (4.018)	0.854 (3.806)	0.807 (2.554)	-0.047 (8.276)	0.387 (0.151)	0.865 (9.078)	0.569 (1.105)	1.872565	0.095607	0.134975
**Fractal dimension zone c**	0.881 (0.068)	0.880 (0.068)	0.885 (0.060)	0.888 (0.066)	0.917 (0.077)	0.889 (0.068)	0.889 (0.068)	3.130809	0.007957	0.015483
**Vessel density zone c**	0.014 (0.003)	0.014 (0.003)	0.015 (0.002)	0.015 (0.002)	0.015 (0.002)	0.015 (0.003)	0.015 (0.003)	15.51137	3.39E-15	2.44E-14
**Average width zone c**	46.304 (4.219)	46.340 (4.236)	45.514 (4.331)	45.619 (3.814)	48.285 (4.041)	46.758 (4.047)	45.896 (3.542)	3.991022	0.001289	0.003438
**Distance tortuosity zone c**	2.408 (1.175)	2.407 (1.170)	2.432 (1.128)	2.439 (1.397)	2.615 (1.220)	2.208 (1.106)	2.430 (1.195)	0.649171	0.662151	0.701101
**Squared curvature tortuosity zone c**	16.351 (18.715)	16.412 (18.784)	16.438 (16.998)	15.880 (20.765)	15.937 (18.226)	13.229 (17.625)	16.792 (16.695)	0.426594	0.830436	0.830436
**Tortuosity density zone c**	0.469 (0.044)	0.470 (0.045)	0.472 (0.044)	0.463 (0.040)	0.473 (0.046)	0.466 (0.046)	0.462 (0.047)	1.651687	0.142733	0.181319
**Artery Fractal dimension zone c**	0.759 (0.059)	0.758 (0.059)	0.756 (0.052)	0.760 (0.058)	0.788 (0.070)	0.764 (0.058)	0.767 (0.058)	2.415689	0.033846	0.055384
**Artery Vessel density zone c**	0.006 (0.001)	0.006 (0.001)	0.006 (0.001)	0.006 (0.001)	0.006 (0.001)	0.006 (0.001)	0.006 (0.001)	10.47235	5.01E-10	2.78E-09
**Artery Average width zone c**	44.362 (4.530)	44.310 (4.562)	44.772 (4.540)	44.796 (4.172)	46.298 (3.275)	44.953 (4.277)	44.700 (3.737)	2.328688	0.04014	0.062828
**Artery Distance tortuosity zone c**	3.163 (2.428)	3.159 (2.432)	3.364 (2.748)	2.937 (2.000)	3.348 (2.336)	3.011 (2.273)	3.339 (2.450)	0.733937	0.597911	0.652267
**Artery Squared curvature tortuosity zone c**	30.347 (47.125)	30.480 (47.849)	33.218 (47.713)	24.009 (33.314)	23.393 (29.200)	25.419 (30.145)	34.011 (44.350)	1.1876	0.312437	0.357071
**Artery Tortuosity density zone c**	0.515 (0.058)	0.516 (0.058)	0.512 (0.055)	0.504 (0.055)	0.501 (0.065)	0.506 (0.068)	0.505 (0.058)	2.712953	0.01871	0.032857
**CRAE Hubbard zone c**	107.816 (11.654)	107.553 (11.639)	109.364 (11.990)	110.666 (11.187)	114.078 (9.224)	112.037 (11.940)	110.264 (11.628)	8.001467	1.57E-07	6.66E-07
**CRAE Knudtson zone c**	96.146 (10.588)	95.903 (10.572)	97.603 (10.935)	98.828 (10.151)	101.753 (8.612)	100.096 (10.768)	98.361 (10.582)	8.260959	8.64E-08	3.89E-07
**Vein Fractal dimension zone c**	0.067 (1.180)	0.062 (1.189)	0.155 (1.174)	0.166 (0.857)	-0.374 (1.415)	0.060 (1.311)	0.096 (1.104)	1.396309	0.222179	0.262244
**Vein Vessel density zone c**	0.007 (0.002)	0.007 (0.002)	0.008 (0.002)	0.008 (0.001)	0.008 (0.002)	0.008 (0.002)	0.008 (0.002)	40.81442	2.26E-41	5.43E-40

Feature	Overall	White	Black	Asian	Chinese	Mixed	Others	F_stat	p_value	FDR_p
**Vein Average width zone c**	49.834 (5.975)	49.865 (6.045)	49.194 (4.967)	48.991 (4.988)	52.759 (5.567)	50.355 (5.299)	49.493 (5.395)	2.595314	0.02371	0.039788
**Vein Distance tortuosity zone c**	2.329 (1.931)	2.309 (1.921)	2.580 (1.932)	2.696 (1.998)	2.322 (1.314)	2.247 (2.328)	2.470 (2.079)	1.824565	0.104457	0.144633
**Vein Squared curvature tortuosity zone c**	15.378 (29.302)	14.980 (28.115)	19.982 (27.982)	21.562 (46.487)	16.513 (19.418)	16.576 (42.363)	16.341 (35.432)	2.335293	0.039641	0.062828
**Vein Tortuosity density zone c**	0.498 (0.062)	0.498 (0.062)	0.508 (0.059)	0.507 (0.054)	0.509 (0.063)	0.491 (0.061)	0.492 (0.059)	1.878978	0.094502	0.134975
**CRVE Hubbard zone c**	137.358 (13.472)	136.841 (13.343)	144.801 (14.015)	141.831 (12.928)	146.132 (14.570)	140.347 (12.244)	142.138 (14.076)	19.15731	5.99E-19	5.39E-18
**CRVE Knudtson zone c**	149.355 (15.710)	148.754 (15.559)	158.103 (16.335)	154.587 (14.954)	158.969 (16.887)	152.869 (14.788)	154.856 (16.553)	18.92702	1.04E-18	8.29E-18
**AVR Hubbard zone c**	0.575 (1.408)	0.573 (1.383)	0.776 (3.132)	0.539 (0.063)	0.547 (0.052)	0.543 (0.064)	0.531 (0.068)	0.608854	0.693163	0.712968
**AVR Knudtson zone c**	0.469 (1.040)	0.467 (1.034)	0.596 (2.097)	0.443 (0.054)	0.449 (0.045)	0.446 (0.057)	0.436 (0.058)	0.45583	0.809302	0.820701

CDR = Cup-to-disc ratio, CRAE = Central Retinal Artery Equivalent, CRVE = Central Retinal Vein Equivalent, AVR = Artery-Vein Ratio
