# Supplementary material for: Ethnicity-Stratified Normative Retinal Vascular Features from the UK Biobank Using Deep Learning
Source: Ophthalmol Sci. 2026 May 8;6(7):101221. doi: 10.1016/j.xops.2026.101221 (PMC13260094; doi:10.1016/j.xops.2026.101221)
Supplement: Table S5 [file mmc5.pdf]

**Table S5. Associations of the Retinal Morphometric Features with Ethnicity, Age and Sex**

| Feature | Index | Beta | p value | FDR p |
|---|---|---|---|---|
| Disc height | C(Age_group)[T.50-59] | -15.28836215 | 0.00100029 | 0.003928413 |
| | C(Age_group)[T.60-69] | -41.34522389 | 4.24E-15 | 5.72E-14 |
| | C(Age_group)[T.70+] | -121.7541678 | 4.50E-06 | 2.60E-05 |
| | C(Sex)[T.Male] | -12.42265472 | 0.00181091 | 0.0068225 |
| | C(Ethnicity)[T.Asian] | 89.1542722 | 1.34E-13 | 1.50E-12 |
| | C(Ethnicity)[T.Black] | 117.8863599 | 1.36E-20 | 2.52E-19 |
| | C(Ethnicity)[T.Chinese] | 141.5993986 | 1.28E-07 | 8.63E-07 |
| | C(Ethnicity)[T.Mixed] | 36.7394753 | 0.053480555 | 0.122457243 |
| | C(Ethnicity)[T.Others] | 62.5462406 | 2.68E-05 | 0.000138775 |
| Disc width | C(Age_group)[T.50-59] | -8.006852516 | 0.086768162 | 0.183145828 |
| | C(Age_group)[T.60-69] | -23.45530315 | 9.39E-06 | 5.24E-05 |
| | C(Age_group)[T.70+] | -46.19033539 | 0.083604297 | 0.178209159 |
| | C(Sex)[T.Male] | 8.272650246 | 0.038970996 | 0.094936862 |
| | C(Ethnicity)[T.Asian] | 39.34814486 | 0.001147289 | 0.004478575 |
| | C(Ethnicity)[T.Black] | 23.01575232 | 0.070187788 | 0.154699614 |
| | C(Ethnicity)[T.Chinese] | 63.9801626 | 0.017649541 | 0.048461452 |
| | C(Ethnicity)[T.Mixed] | 3.987229663 | 0.835036785 | 0.907814802 |
| | C(Ethnicity)[T.Others] | -3.896763608 | 0.794769542 | 0.890229576 |
| Cup height | C(Age_group)[T.50-59] | -7.930062649 | 0.030554178 | 0.077643558 |
| | C(Age_group)[T.60-69] | -17.27527429 | 3.16E-05 | 0.000162512 |
| | C(Age_group)[T.70+] | -33.93671907 | 0.105031885 | 0.214027238 |
| | C(Sex)[T.Male] | -6.946061919 | 0.027083358 | 0.069642922 |
| | C(Ethnicity)[T.Asian] | 71.70101607 | 4.65E-14 | 5.38E-13 |
| | C(Ethnicity)[T.Black] | 101.4959596 | 3.63E-24 | 7.34E-23 |
| | C(Ethnicity)[T.Chinese] | 105.2397295 | 6.59E-07 | 4.06E-06 |
| | C(Ethnicity)[T.Mixed] | 14.75017235 | 0.325951981 | 0.511817416 |
| | C(Ethnicity)[T.Others] | 39.83755916 | 0.000700426 | 0.002836725 |
| Cup width | C(Age_group)[T.50-59] | -8.803097089 | 0.020078322 | 0.053542191 |
| | C(Age_group)[T.60-69] | -20.00387522 | 3.08E-06 | 1.80E-05 |
| | C(Age_group)[T.70+] | -44.82528833 | 0.038162514 | 0.094027791 |
| | C(Sex)[T.Male] | 4.521452365 | 0.163498086 | 0.305322074 |

| Feature | Index | Beta | p value | FDR p |
|---|---|---|---|---|
| | C(Ethnicity)[T.Asian] | 61.84600658 | 2.91E-10 | 2.45E-09 |
| | C(Ethnicity)[T.Black] | 80.52597039 | 5.98E-15 | 7.91E-14 |
| | C(Ethnicity)[T.Chinese] | 90.28873033 | 3.58E-05 | 0.000181342 |
| | C(Ethnicity)[T.Mixed] | 11.87596742 | 0.443754059 | 0.631983802 |
| | C(Ethnicity)[T.Others] | 26.27089154 | 0.030388021 | 0.077525345 |
| CDR vertical | C(Age_group)[T.50-59] | -0.002036031 | 0.213419986 | 0.376828749 |
| | C(Age_group)[T.60-69] | -0.00469372 | 0.011267641 | 0.034279021 |
| | C(Age_group)[T.70+] | 0.019021722 | 0.041820061 | 0.100712839 |
| | C(Sex)[T.Male] | -0.000360035 | 0.797397237 | 0.890229576 |
| | C(Ethnicity)[T.Asian] | 0.016729326 | 7.86E-05 | 0.00039186 |
| | C(Ethnicity)[T.Black] | 0.024737715 | 2.80E-08 | 1.99E-07 |
| | C(Ethnicity)[T.Chinese] | 0.034388437 | 0.000270175 | 0.001199134 |
| | C(Ethnicity)[T.Mixed] | -0.003485587 | 0.603027805 | 0.761719332 |
| | C(Ethnicity)[T.Others] | 0.003577368 | 0.495125629 | 0.681191949 |
| CDR horizontal | C(Age_group)[T.50-59] | -0.005274159 | 0.002472162 | 0.009154061 |
| | C(Age_group)[T.60-69] | -0.011804992 | 2.23E-09 | 1.72E-08 |
| | C(Age_group)[T.70+] | -0.014147675 | 0.15498464 | 0.294516265 |
| | C(Sex)[T.Male] | 0.000622503 | 0.676709662 | 0.825815181 |
| | C(Ethnicity)[T.Asian] | 0.027114777 | 1.89E-09 | 1.49E-08 |
| | C(Ethnicity)[T.Black] | 0.04325716 | 8.76E-20 | 1.58E-18 |
| | C(Ethnicity)[T.Chinese] | 0.050213299 | 5.92E-07 | 3.73E-06 |
| | C(Ethnicity)[T.Mixed] | 0.005220792 | 0.464341073 | 0.649034721 |
| | C(Ethnicity)[T.Others] | 0.016621862 | 0.00291535 | 0.010437276 |
| Fractal dimension | C(Age_group)[T.50-59] | -0.01503404 | 2.37E-13 | 2.60E-12 |
| | C(Age_group)[T.60-69] | -0.041348998 | 4.49E-71 | 2.91E-69 |
| | C(Age_group)[T.70+] | -0.063351757 | 4.42E-08 | 3.05E-07 |
| | C(Sex)[T.Male] | 0.003324172 | 0.056689627 | 0.12889431 |
| | C(Ethnicity)[T.Asian] | 0.002887711 | 0.590500849 | 0.751757466 |
| | C(Ethnicity)[T.Black] | -0.002113587 | 0.707669515 | 0.844511686 |
| | C(Ethnicity)[T.Chinese] | 0.043039819 | 0.000337096 | 0.001456253 |
| | C(Ethnicity)[T.Mixed] | 0.003297503 | 0.696768615 | 0.8392306 |
| | C(Ethnicity)[T.Others] | -0.000131613 | 0.984178426 | 0.992859331 |

| Feature | Index | Beta | p value | FDR p |
|---|---|---|---|---|
| Vessel density | C(Age_group)[T.50-59] | -0.002745091 | 4.50E-52 | 1.71E-50 |
| | C(Age_group)[T.60-69] | -0.006435213 | 5.69E-210 | 1.84E-207 |
| | C(Age_group)[T.70+] | -0.011020602 | 2.11E-27 | 4.89E-26 |
| | C(Sex)[T.Male] | 0.000379856 | 0.012828897 | 0.037615952 |
| | C(Ethnicity)[T.Asian] | 0.000254832 | 0.587336645 | 0.749201074 |
| | C(Ethnicity)[T.Black] | -0.000728988 | 0.139410436 | 0.269915375 |
| | C(Ethnicity)[T.Chinese] | 0.001857017 | 0.076992136 | 0.166859211 |
| | C(Ethnicity)[T.Mixed] | 0.001094072 | 0.139539584 | 0.269915375 |
| | C(Ethnicity)[T.Others] | 0.001322781 | 0.022765293 | 0.059724333 |
| Average width | C(Age_group)[T.50-59] | -0.071257261 | 0.464740911 | 0.649034721 |
| | C(Age_group)[T.60-69] | -0.108762538 | 0.318661025 | 0.506676065 |
| | C(Age_group)[T.70+] | -0.203230256 | 0.711888304 | 0.846246633 |
| | C(Sex)[T.Male] | -0.747309866 | 2.84E-19 | 4.98E-18 |
| | C(Ethnicity)[T.Asian] | -0.439310447 | 0.085472518 | 0.181000626 |
| | C(Ethnicity)[T.Black] | -0.589438754 | 0.02803648 | 0.071808849 |
| | C(Ethnicity)[T.Chinese] | 1.210113577 | 0.034139983 | 0.084761337 |
| | C(Ethnicity)[T.Mixed] | 0.257407103 | 0.522752726 | 0.707189492 |
| | C(Ethnicity)[T.Others] | -0.211085447 | 0.50399325 | 0.686672799 |
| Distance tortuosity | C(Age_group)[T.50-59] | 0.100163811 | 3.03E-06 | 1.80E-05 |
| | C(Age_group)[T.60-69] | 0.206261719 | 9.93E-18 | 1.65E-16 |
| | C(Age_group)[T.70+] | 0.162406264 | 0.179649273 | 0.329953417 |
| | C(Sex)[T.Male] | -0.096553168 | 1.27E-07 | 8.63E-07 |
| | C(Ethnicity)[T.Asian] | -0.125697811 | 0.025273803 | 0.065248703 |
| | C(Ethnicity)[T.Black] | -0.142720466 | 0.015589769 | 0.044114281 |
| | C(Ethnicity)[T.Chinese] | 0.037207579 | 0.767070575 | 0.872038128 |
| | C(Ethnicity)[T.Mixed] | -0.20883042 | 0.01842045 | 0.049735216 |
| | C(Ethnicity)[T.Others] | -0.138189849 | 0.046725303 | 0.108523284 |
| Squared curvature tortuosity | C(Age_group)[T.50-59] | 1.530048459 | 0.000227555 | 0.001031158 |
| | C(Age_group)[T.60-69] | 3.67391164 | 2.89E-15 | 4.16E-14 |
| | C(Age_group)[T.70+] | 6.847086116 | 0.00347214 | 0.012096486 |
| | C(Sex)[T.Male] | -1.532816822 | 1.46E-05 | 7.86E-05 |
| | C(Ethnicity)[T.Asian] | -1.902297823 | 0.080176946 | 0.172606847 |

| Feature | Index | Beta | p value | FDR p |
|---|---|---|---|---|
| | C(Ethnicity)[T.Black] | -2.342753076 | 0.040223792 | 0.097257526 |
| | C(Ethnicity)[T.Chinese] | -1.320527616 | 0.58697644 | 0.749201074 |
| | C(Ethnicity)[T.Mixed] | -4.034908397 | 0.01860757 | 0.049978081 |
| | C(Ethnicity)[T.Others] | -2.508465342 | 0.062111934 | 0.138311112 |
| Tortuosity density | C(Age_group)[T.50-59] | -0.000565506 | 0.60017082 | 0.759591194 |
| | C(Age_group)[T.60-69] | -0.004345742 | 0.000320627 | 0.001394405 |
| | C(Age_group)[T.70+] | -0.001078606 | 0.859444392 | 0.920153241 |
| | C(Sex)[T.Male] | -0.000817217 | 0.373811582 | 0.562018341 |
| | C(Ethnicity)[T.Asian] | -0.006749488 | 0.016994996 | 0.047063066 |
| | C(Ethnicity)[T.Black] | -2.10E-05 | 0.994346436 | 0.996099388 |
| | C(Ethnicity)[T.Chinese] | 0.004550725 | 0.471635486 | 0.654432109 |
| | C(Ethnicity)[T.Mixed] | -0.003495366 | 0.433019493 | 0.620881864 |
| | C(Ethnicity)[T.Others] | -0.006167306 | 0.077796177 | 0.168039743 |
| Artery Fractal dimension | C(Age_group)[T.50-59] | -0.013732585 | 9.45E-15 | 1.23E-13 |
| | C(Age_group)[T.60-69] | -0.037896877 | 1.46E-79 | 1.18E-77 |
| | C(Age_group)[T.70+] | -0.0596323 | 2.46E-09 | 1.86E-08 |
| | C(Sex)[T.Male] | 0.001118999 | 0.457578762 | 0.643190971 |
| | C(Ethnicity)[T.Asian] | 0.001632114 | 0.724733782 | 0.852318494 |
| | C(Ethnicity)[T.Black] | -0.0068542 | 0.159165562 | 0.298954447 |
| | C(Ethnicity)[T.Chinese] | 0.037778695 | 0.000269115 | 0.001199134 |
| | C(Ethnicity)[T.Mixed] | 0.004537356 | 0.534711693 | 0.714418922 |
| | C(Ethnicity)[T.Others] | -3.91E-05 | 0.994562197 | 0.996099388 |
| Artery Vessel density | C(Age_group)[T.50-59] | -0.001454916 | 3.46E-62 | 1.87E-60 |
| | C(Age_group)[T.60-69] | -0.003256327 | 5.87E-229 | 3.80E-226 |
| | C(Age_group)[T.70+] | -0.005546069 | 1.29E-29 | 3.09E-28 |
| | C(Sex)[T.Male] | -0.000148317 | 0.044110494 | 0.103940363 |
| | C(Ethnicity)[T.Asian] | 0.000286636 | 0.206045346 | 0.365801052 |
| | C(Ethnicity)[T.Black] | -0.000635398 | 0.007625101 | 0.02446072 |
| | C(Ethnicity)[T.Chinese] | 0.001017573 | 0.044710218 | 0.104971816 |
| | C(Ethnicity)[T.Mixed] | 0.00089767 | 0.012039997 | 0.035788615 |
| | C(Ethnicity)[T.Others] | 0.000739993 | 0.008313591 | 0.026537965 |
| Artery Average width | C(Age_group)[T.50-59] | -0.616702797 | 2.76E-09 | 2.05E-08 |

| Feature | Index | Beta | p value | FDR p |
|---|---|---|---|---|
| | C(Age_group)[T.60-69] | -0.912300753 | 4.08E-15 | 5.63E-14 |
| | C(Age_group)[T.70+] | -1.177767558 | 0.044050805 | 0.103940363 |
| | C(Sex)[T.Male] | -0.840819996 | 2.15E-21 | 4.21E-20 |
| | C(Ethnicity)[T.Asian] | 0.005329061 | 0.984336887 | 0.992859331 |
| | C(Ethnicity)[T.Black] | -0.044888662 | 0.874894287 | 0.931633912 |
| | C(Ethnicity)[T.Chinese] | 1.59546753 | 0.008593181 | 0.027295986 |
| | C(Ethnicity)[T.Mixed] | 0.352868682 | 0.409724286 | 0.600681759 |
| | C(Ethnicity)[T.Others] | 0.179927718 | 0.591991259 | 0.752177129 |
| Artery Distance tortuosity | C(Age_group)[T.50-59] | 0.160452535 | 0.009586491 | 0.029865606 |
| | C(Age_group)[T.60-69] | 0.323516878 | 3.09E-06 | 1.80E-05 |
| | C(Age_group)[T.70+] | 0.027638377 | 0.936989039 | 0.965292364 |
| | C(Sex)[T.Male] | -0.144215356 | 0.006263608 | 0.020603137 |
| | C(Ethnicity)[T.Asian] | -0.591680717 | 0.000268122 | 0.001199134 |
| | C(Ethnicity)[T.Black] | -0.205607966 | 0.227717404 | 0.394547801 |
| | C(Ethnicity)[T.Chinese] | -0.006034968 | 0.98673057 | 0.992859331 |
| | C(Ethnicity)[T.Mixed] | -0.444988783 | 0.08206497 | 0.175505282 |
| | C(Ethnicity)[T.Others] | -0.21666151 | 0.280358449 | 0.462617517 |
| Artery Squared curvature tortuosity | C(Age_group)[T.50-59] | 6.075351333 | 0.001507649 | 0.005713195 |
| | C(Age_group)[T.60-69] | 12.13026089 | 1.54E-08 | 1.11E-07 |
| | C(Age_group)[T.70+] | 12.00437798 | 0.266586072 | 0.442943013 |
| | C(Sex)[T.Male] | -2.208937095 | 0.175384694 | 0.323787127 |
| | C(Ethnicity)[T.Asian] | -13.55426965 | 0.006894921 | 0.022565195 |
| | C(Ethnicity)[T.Black] | -1.97670115 | 0.707470556 | 0.844511686 |
| | C(Ethnicity)[T.Chinese] | -2.129994624 | 0.84936236 | 0.914723684 |
| | C(Ethnicity)[T.Mixed] | -6.199596554 | 0.433084263 | 0.620881864 |
| | C(Ethnicity)[T.Others] | -2.200147383 | 0.72280349 | 0.85159393 |
| Artery Tortuosity density | C(Age_group)[T.50-59] | -5.44E-05 | 0.96837585 | 0.985889932 |
| | C(Age_group)[T.60-69] | -0.004991859 | 0.001162975 | 0.004512622 |
| | C(Age_group)[T.70+] | 0.000994468 | 0.897920694 | 0.939988061 |
| | C(Sex)[T.Male] | -0.002297953 | 0.049439406 | 0.114009734 |
| | C(Ethnicity)[T.Asian] | -0.010596145 | 0.003240359 | 0.011537103 |
| | C(Ethnicity)[T.Black] | -0.007543453 | 0.045959165 | 0.107514582 |

| Feature | Index | Beta | p value | FDR p |
|---|---|---|---|---|
| | C(Ethnicity)[T.Chinese] | -0.002944659 | 0.714371197 | 0.846275203 |
| | C(Ethnicity)[T.Mixed] | -0.008686213 | 0.125808119 | 0.247792283 |
| | C(Ethnicity)[T.Others] | -0.014274494 | 0.001343421 | 0.005120805 |
| Vein Fractal dimension | C(Age_group)[T.50-59] | -0.014519485 | 1.15E-14 | 1.41E-13 |
| | C(Age_group)[T.60-69] | -0.039052015 | 2.67E-75 | 1.92E-73 |
| | C(Age_group)[T.70+] | -0.058296469 | 3.86E-08 | 2.69E-07 |
| | C(Sex)[T.Male] | 0.004644579 | 0.00366411 | 0.012629485 |
| | C(Ethnicity)[T.Asian] | 0.006569903 | 0.18148645 | 0.329953417 |
| | C(Ethnicity)[T.Black] | 0.004600404 | 0.373015372 | 0.562018341 |
| | C(Ethnicity)[T.Chinese] | 0.041769266 | 0.000146332 | 0.000677306 |
| | C(Ethnicity)[T.Mixed] | 0.004478726 | 0.563470065 | 0.727347813 |
| | C(Ethnicity)[T.Others] | 0.003790558 | 0.533033369 | 0.714051913 |
| Vein Vessel density | C(Age_group)[T.50-59] | -0.001469551 | 4.61E-48 | 1.42E-46 |
| | C(Age_group)[T.60-69] | -0.003238552 | 3.61E-173 | 7.80E-171 |
| | C(Age_group)[T.70+] | -0.005345937 | 4.08E-21 | 7.78E-20 |
| | C(Sex)[T.Male] | 0.000574329 | 1.73E-11 | 1.70E-10 |
| | C(Ethnicity)[T.Asian] | 0.000893085 | 0.000663361 | 0.002703508 |
| | C(Ethnicity)[T.Black] | 0.000924249 | 0.000795582 | 0.003162803 |
| | C(Ethnicity)[T.Chinese] | 0.002111896 | 0.000318315 | 0.001394405 |
| | C(Ethnicity)[T.Mixed] | 0.000693515 | 0.093532485 | 0.193639138 |
| | C(Ethnicity)[T.Others] | 0.001441522 | 8.93E-06 | 5.03E-05 |
| Vein Average width | C(Age_group)[T.50-59] | 0.043854932 | 0.728281274 | 0.853320758 |
| | C(Age_group)[T.60-69] | 0.632979826 | 7.54E-06 | 4.32E-05 |
| | C(Age_group)[T.70+] | 1.6172252 | 0.023280014 | 0.060828423 |
| | C(Sex)[T.Male] | -0.535854601 | 6.37E-07 | 3.97E-06 |
| | C(Ethnicity)[T.Asian] | -0.524721823 | 0.112714869 | 0.225544081 |
| | C(Ethnicity)[T.Black] | -0.704976113 | 0.042483927 | 0.101585182 |
| | C(Ethnicity)[T.Chinese] | 1.695011668 | 0.021958952 | 0.058317216 |
| | C(Ethnicity)[T.Mixed] | -0.155146204 | 0.766132842 | 0.872038128 |
| | C(Ethnicity)[T.Others] | -0.545213725 | 0.182662281 | 0.330628933 |
| Vein Distance tortuosity | C(Age_group)[T.50-59] | 0.132096194 | 2.49E-05 | 0.000130096 |
| | C(Age_group)[T.60-69] | 0.260038437 | 1.30E-13 | 1.48E-12 |

| Feature | Index | Beta | p value | FDR p |
|---|---|---|---|---|
| | C(Age_group)[T.70+] | 0.198589929 | 0.261304127 | 0.43640483 |
| | C(Sex)[T.Male] | -0.028061394 | 0.29270973 | 0.479264067 |
| | C(Ethnicity)[T.Asian] | 0.038385215 | 0.63992949 | 0.798987109 |
| | C(Ethnicity)[T.Black] | -0.075252457 | 0.382588456 | 0.573882683 |
| | C(Ethnicity)[T.Chinese] | 0.005592404 | 0.975684796 | 0.989426836 |
| | C(Ethnicity)[T.Mixed] | -0.142512295 | 0.270728821 | 0.448675898 |
| | C(Ethnicity)[T.Others] | 0.058888758 | 0.561721688 | 0.726538231 |
| Vein Squared curvature tortuosity | C(Age_group)[T.50-59] | 2.399117491 | 0.000225219 | 0.001027762 |
| | C(Age_group)[T.60-69] | 4.520788174 | 5.42E-10 | 4.45E-09 |
| | C(Age_group)[T.70+] | 0.789285239 | 0.829708686 | 0.90361551 |
| | C(Sex)[T.Male] | -0.24855037 | 0.653459552 | 0.806802656 |
| | C(Ethnicity)[T.Asian] | 1.039391279 | 0.541738438 | 0.717886519 |
| | C(Ethnicity)[T.Black] | -1.504703765 | 0.400337419 | 0.58972569 |
| | C(Ethnicity)[T.Chinese] | -3.254716727 | 0.392841541 | 0.583856236 |
| | C(Ethnicity)[T.Mixed] | -3.282858241 | 0.221648637 | 0.38713832 |
| | C(Ethnicity)[T.Others] | 0.79950498 | 0.704307204 | 0.844511686 |
| Vein Tortuosity density | C(Age_group)[T.50-59] | 4.73E-05 | 0.969154147 | 0.985889932 |
| | C(Age_group)[T.60-69] | -0.005366985 | 8.83E-05 | 0.000436637 |
| | C(Age_group)[T.70+] | -0.00238652 | 0.729536574 | 0.853320758 |
| | C(Sex)[T.Male] | -0.000102747 | 0.921397556 | 0.957666183 |
| | C(Ethnicity)[T.Asian] | -0.001457894 | 0.649084692 | 0.806802656 |
| | C(Ethnicity)[T.Black] | 0.004118246 | 0.22105397 | 0.38713832 |
| | C(Ethnicity)[T.Chinese] | 0.01614572 | 0.024245376 | 0.063096401 |
| | C(Ethnicity)[T.Mixed] | -0.000889065 | 0.860309275 | 0.920153241 |
| | C(Ethnicity)[T.Others] | -0.002439024 | 0.538204806 | 0.716712119 |
| Fractal dimension zone b | C(Age_group)[T.50-59] | -0.010439032 | 8.83E-09 | 6.50E-08 |
| | C(Age_group)[T.60-69] | -0.03033383 | 1.16E-48 | 3.75E-47 |
| | C(Age_group)[T.70+] | -0.035573259 | 0.000591989 | 0.00244337 |
| | C(Sex)[T.Male] | 0.002147429 | 0.166924134 | 0.31082425 |
| | C(Ethnicity)[T.Asian] | -0.000968697 | 0.836366415 | 0.907814802 |
| | C(Ethnicity)[T.Black] | -0.004806067 | 0.329444273 | 0.51440937 |
| | C(Ethnicity)[T.Chinese] | 0.02183267 | 0.036759507 | 0.090916643 |

| Feature | Index | Beta | p value | FDR p |
|---|---|---|---|---|
| | C(Ethnicity)[T.Mixed] | -0.004147101 | 0.57643261 | 0.739660062 |
| | C(Ethnicity)[T.Others] | -0.004376694 | 0.451164945 | 0.636938746 |
| Vessel density zone b | C(Age_group)[T.50-59] | -0.000200353 | 4.06E-14 | 4.79E-13 |
| | C(Age_group)[T.60-69] | -0.000506401 | 7.89E-63 | 4.65E-61 |
| | C(Age_group)[T.70+] | -0.00076705 | 3.90E-07 | 2.50E-06 |
| | C(Sex)[T.Male] | 1.51E-05 | 0.504407797 | 0.686672799 |
| | C(Ethnicity)[T.Asian] | 5.82E-05 | 0.395156884 | 0.584615665 |
| | C(Ethnicity)[T.Black] | 4.27E-05 | 0.553029372 | 0.722065274 |
| | C(Ethnicity)[T.Chinese] | 7.32E-05 | 0.63125975 | 0.791211446 |
| | C(Ethnicity)[T.Mixed] | 9.66E-05 | 0.372300884 | 0.562018341 |
| | C(Ethnicity)[T.Others] | 4.99E-05 | 0.555934229 | 0.722065274 |
| Average width zone b | C(Age_group)[T.50-59] | -0.385391245 | 0.006965678 | 0.022682208 |
| | C(Age_group)[T.60-69] | -0.623151207 | 0.000116151 | 0.000545165 |
| | C(Age_group)[T.70+] | -1.944017092 | 0.017137538 | 0.047255849 |
| | C(Sex)[T.Male] | -0.747161512 | 1.09E-09 | 8.69E-09 |
| | C(Ethnicity)[T.Asian] | -0.001071638 | 0.997685649 | 0.997685649 |
| | C(Ethnicity)[T.Black] | -0.51838885 | 0.181779892 | 0.329953417 |
| | C(Ethnicity)[T.Chinese] | 2.819470845 | 0.000620031 | 0.002542914 |
| | C(Ethnicity)[T.Mixed] | 0.719430687 | 0.218656136 | 0.383981507 |
| | C(Ethnicity)[T.Others] | 0.025727953 | 0.955159994 | 0.9780887 |
| Distance tortuosity zone b | C(Age_group)[T.50-59] | -0.048687595 | 0.095166143 | 0.195770351 |
| | C(Age_group)[T.60-69] | -0.118861849 | 0.000320434 | 0.001394405 |
| | C(Age_group)[T.70+] | -0.3919467 | 0.018664654 | 0.049978081 |
| | C(Sex)[T.Male] | -0.009289716 | 0.71026033 | 0.846045393 |
| | C(Ethnicity)[T.Asian] | 0.018769127 | 0.803642987 | 0.890663021 |
| | C(Ethnicity)[T.Black] | 0.093483054 | 0.238586756 | 0.410090763 |
| | C(Ethnicity)[T.Chinese] | -0.089498268 | 0.59474186 | 0.7541932 |
| | C(Ethnicity)[T.Mixed] | -0.096373014 | 0.419955674 | 0.608794803 |
| | C(Ethnicity)[T.Others] | -0.039920316 | 0.669386309 | 0.821519561 |
| Squared curvature tortuosity zone b | C(Age_group)[T.50-59] | -0.367685162 | 0.091591452 | 0.190840068 |
| | C(Age_group)[T.60-69] | -0.581603316 | 0.018390895 | 0.049735216 |
| | C(Age_group)[T.70+] | -1.451426999 | 0.243515053 | 0.414167334 |

| Feature | Index | Beta | p value | FDR p |
|---|---|---|---|---|
| | C(Sex)[T.Male] | -0.116357091 | 0.533335071 | 0.714051913 |
| | C(Ethnicity)[T.Asian] | 0.136342668 | 0.80894547 | 0.893142342 |
| | C(Ethnicity)[T.Black] | 2.040227482 | 0.000577873 | 0.002420268 |
| | C(Ethnicity)[T.Chinese] | -1.570105385 | 0.211555292 | 0.374556911 |
| | C(Ethnicity)[T.Mixed] | 0.208470825 | 0.815331958 | 0.895417834 |
| | C(Ethnicity)[T.Others] | -0.714600965 | 0.306219828 | 0.496076121 |
| Tortuosity density zone b | C(Age_group)[T.50-59] | -0.001912524 | 0.240789209 | 0.411692368 |
| | C(Age_group)[T.60-69] | -0.006815677 | 0.000222674 | 0.001023355 |
| | C(Age_group)[T.70+] | -0.015680352 | 0.092181959 | 0.191454837 |
| | C(Sex)[T.Male] | -0.000484057 | 0.729052975 | 0.853320758 |
| | C(Ethnicity)[T.Asian] | -0.002749754 | 0.51453956 | 0.697534801 |
| | C(Ethnicity)[T.Black] | 0.003279453 | 0.459414275 | 0.644373268 |
| | C(Ethnicity)[T.Chinese] | 0.006341364 | 0.500018449 | 0.685448393 |
| | C(Ethnicity)[T.Mixed] | -0.002973275 | 0.656149691 | 0.806802656 |
| | C(Ethnicity)[T.Others] | -0.015834518 | 0.002449596 | 0.009122633 |
| Artery Fractal dimension zone b | C(Age_group)[T.50-59] | -0.009149468 | 3.40E-08 | 2.40E-07 |
| | C(Age_group)[T.60-69] | -0.0260211 | 3.32E-43 | 8.97E-42 |
| | C(Age_group)[T.70+] | -0.036541486 | 0.000112241 | 0.000534234 |
| | C(Sex)[T.Male] | 0.000643111 | 0.650433082 | 0.806802656 |
| | C(Ethnicity)[T.Asian] | -0.00428157 | 0.317634815 | 0.506676065 |
| | C(Ethnicity)[T.Black] | -0.01101268 | 0.014452623 | 0.041256826 |
| | C(Ethnicity)[T.Chinese] | 0.020278662 | 0.033713885 | 0.084321782 |
| | C(Ethnicity)[T.Mixed] | -0.002074539 | 0.759678964 | 0.86921401 |
| | C(Ethnicity)[T.Others] | -0.004987591 | 0.347241295 | 0.535743712 |
| Artery Vessel density zone b | C(Age_group)[T.50-59] | -0.000118406 | 1.05E-17 | 1.70E-16 |
| | C(Age_group)[T.60-69] | -0.000255063 | 6.36E-59 | 2.94E-57 |
| | C(Age_group)[T.70+] | -0.000451602 | 9.92E-09 | 7.22E-08 |
| | C(Sex)[T.Male] | -7.96E-06 | 0.500335015 | 0.685448393 |
| | C(Ethnicity)[T.Asian] | 3.53E-05 | 0.321809991 | 0.509860329 |
| | C(Ethnicity)[T.Black] | -3.73E-05 | 0.319018263 | 0.506676065 |
| | C(Ethnicity)[T.Chinese] | 2.37E-05 | 0.765879662 | 0.872038128 |
| | C(Ethnicity)[T.Mixed] | 6.53E-05 | 0.247084131 | 0.418650914 |

| Feature | Index | Beta | p value | FDR p |
|---|---|---|---|---|
| | C(Ethnicity)[T.Others] | 1.76E-05 | 0.689585914 | 0.83479018 |
| Artery Average width zone b | C(Age_group)[T.50-59] | -1.095773943 | 6.09E-12 | 6.37E-11 |
| | C(Age_group)[T.60-69] | -1.485501203 | 1.85E-16 | 2.79E-15 |
| | C(Age_group)[T.70+] | -1.103749363 | 0.224240262 | 0.390612069 |
| | C(Sex)[T.Male] | -0.936357658 | 7.06E-12 | 7.26E-11 |
| | C(Ethnicity)[T.Asian] | 0.987567989 | 0.016422853 | 0.046069301 |
| | C(Ethnicity)[T.Black] | 0.496201586 | 0.251146619 | 0.421614013 |
| | C(Ethnicity)[T.Chinese] | 2.264648856 | 0.013555668 | 0.039390462 |
| | C(Ethnicity)[T.Mixed] | 0.290401254 | 0.655721637 | 0.806802656 |
| | C(Ethnicity)[T.Others] | 0.549944054 | 0.280568957 | 0.462617517 |
| Artery Distance tortuosity zone b | C(Age_group)[T.50-59] | -0.009775293 | 0.822493642 | 0.897265791 |
| | C(Age_group)[T.60-69] | -0.14890612 | 0.002541259 | 0.009303592 |
| | C(Age_group)[T.70+] | -0.684317103 | 0.005973056 | 0.019848924 |
| | C(Sex)[T.Male] | 0.00656301 | 0.860513679 | 0.920153241 |
| | C(Ethnicity)[T.Asian] | -0.08171258 | 0.46864207 | 0.651673952 |
| | C(Ethnicity)[T.Black] | -0.086384077 | 0.465918842 | 0.649280451 |
| | C(Ethnicity)[T.Chinese] | -0.287158397 | 0.253160081 | 0.42389595 |
| | C(Ethnicity)[T.Mixed] | -0.05907507 | 0.740652155 | 0.86011218 |
| | C(Ethnicity)[T.Others] | -0.215510431 | 0.12279522 | 0.242595435 |
| Artery Squared curvature tortuosity zone b | C(Age_group)[T.50-59] | 0.193617614 | 0.616079672 | 0.775183743 |
| | C(Age_group)[T.60-69] | -1.000612201 | 0.022090611 | 0.058427413 |
| | C(Age_group)[T.70+] | -4.398071199 | 0.046132774 | 0.10753251 |
| | C(Sex)[T.Male] | 0.107517465 | 0.745314438 | 0.861075312 |
| | C(Ethnicity)[T.Asian] | -0.241435444 | 0.809065671 | 0.893142342 |
| | C(Ethnicity)[T.Black] | -0.64554373 | 0.538640127 | 0.716712119 |
| | C(Ethnicity)[T.Chinese] | -3.566521413 | 0.109274093 | 0.221280039 |
| | C(Ethnicity)[T.Mixed] | -1.110525627 | 0.482602525 | 0.666794107 |
| | C(Ethnicity)[T.Others] | -1.792866749 | 0.147426788 | 0.281794189 |
| Artery Tortuosity density zone b | C(Age_group)[T.50-59] | -3.86E-05 | 0.985415768 | 0.992859331 |
| | C(Age_group)[T.60-69] | -0.006993463 | 0.003421788 | 0.011985508 |
| | C(Age_group)[T.70+] | -0.009128361 | 0.448793967 | 0.636938746 |
| | C(Sex)[T.Male] | -0.003643207 | 0.044038022 | 0.103940363 |

| Feature | Index | Beta | p value | FDR p |
|---|---|---|---|---|
| | C(Ethnicity)[T.Asian] | -0.003215063 | 0.556034833 | 0.722065274 |
| | C(Ethnicity)[T.Black] | -0.001918968 | 0.738047809 | 0.858626535 |
| | C(Ethnicity)[T.Chinese] | -0.011633903 | 0.339120858 | 0.526979174 |
| | C(Ethnicity)[T.Mixed] | -0.009157508 | 0.289441575 | 0.476035889 |
| | C(Ethnicity)[T.Others] | -0.019888786 | 0.003285062 | 0.011591393 |
| CRAE Hubbard zone b | C(Age_group)[T.50-59] | -4.30639649 | 2.31E-11 | 2.17E-10 |
| | C(Age_group)[T.60-69] | -7.752291561 | 5.44E-26 | 1.18E-24 |
| | C(Age_group)[T.70+] | -19.86397027 | 1.73E-07 | 1.13E-06 |
| | C(Sex)[T.Male] | -0.18549954 | 0.737257327 | 0.858626535 |
| | C(Ethnicity)[T.Asian] | -0.983421871 | 0.55257135 | 0.722065274 |
| | C(Ethnicity)[T.Black] | -4.17362752 | 0.016906748 | 0.047019624 |
| | C(Ethnicity)[T.Chinese] | 1.570345545 | 0.673239064 | 0.823130025 |
| | C(Ethnicity)[T.Mixed] | 3.061298251 | 0.240167775 | 0.411692368 |
| | C(Ethnicity)[T.Others] | 0.582976707 | 0.777896901 | 0.88114123 |
| CRAE Knudtson zone b | C(Age_group)[T.50-59] | -3.906632203 | 8.97E-12 | 8.94E-11 |
| | C(Age_group)[T.60-69] | -7.001302747 | 9.00E-27 | 2.01E-25 |
| | C(Age_group)[T.70+] | -17.52896762 | 2.12E-07 | 1.37E-06 |
| | C(Sex)[T.Male] | -0.159687861 | 0.745240278 | 0.861075312 |
| | C(Ethnicity)[T.Asian] | -0.855766119 | 0.560939011 | 0.726538231 |
| | C(Ethnicity)[T.Black] | -3.975527938 | 0.010473229 | 0.03216423 |
| | C(Ethnicity)[T.Chinese] | 1.181570679 | 0.721103537 | 0.851138601 |
| | C(Ethnicity)[T.Mixed] | 2.674865676 | 0.248235958 | 0.41889818 |
| | C(Ethnicity)[T.Others] | 0.287617133 | 0.875591732 | 0.931633912 |
| Vein Fractal dimension zone b | C(Age_group)[T.50-59] | -0.009866083 | 2.43E-09 | 1.85E-08 |
| | C(Age_group)[T.60-69] | -0.027732273 | 5.58E-49 | 1.90E-47 |
| | C(Age_group)[T.70+] | -0.02878473 | 0.00227913 | 0.008536856 |
| | C(Sex)[T.Male] | 0.002074225 | 0.142843411 | 0.274666262 |
| | C(Ethnicity)[T.Asian] | 0.006184339 | 0.147854975 | 0.281794189 |
| | C(Ethnicity)[T.Black] | 0.007096203 | 0.114029172 | 0.227248539 |
| | C(Ethnicity)[T.Chinese] | 0.023714333 | 0.012790004 | 0.037615952 |
| | C(Ethnicity)[T.Mixed] | -0.002324561 | 0.731098195 | 0.853606541 |
| | C(Ethnicity)[T.Others] | 0.00213557 | 0.68655868 | 0.83357854 |

| Feature | Index | Beta | p value | FDR p |
|---|---|---|---|---|
| Vein Vessel density zone b | C(Age_group)[T.50-59] | -0.000104812 | 3.17E-13 | 3.42E-12 |
| | C(Age_group)[T.60-69] | -0.000246178 | 5.59E-51 | 2.01E-49 |
| | C(Age_group)[T.70+] | -0.000318226 | 0.000104385 | 0.000504789 |
| | C(Sex)[T.Male] | 5.21E-06 | 0.67188329 | 0.823025278 |
| | C(Ethnicity)[T.Asian] | 0.00016311 | 1.14E-05 | 6.27E-05 |
| | C(Ethnicity)[T.Black] | 0.00025235 | 1.08E-10 | 9.56E-10 |
| | C(Ethnicity)[T.Chinese] | 0.000208783 | 0.011675921 | 0.035267445 |
| | C(Ethnicity)[T.Mixed] | 0.000114465 | 0.051565944 | 0.118491956 |
| | C(Ethnicity)[T.Others] | 0.000177699 | 0.000112948 | 0.000534234 |
| Vein Average width zone b | C(Age_group)[T.50-59] | -0.222490119 | 0.266299373 | 0.442943013 |
| | C(Age_group)[T.60-69] | 0.383188543 | 0.090737548 | 0.189670746 |
| | C(Age_group)[T.70+] | -1.065293715 | 0.351312603 | 0.536911715 |
| | C(Sex)[T.Male] | -0.55358983 | 0.001256791 | 0.004847624 |
| | C(Ethnicity)[T.Asian] | -0.069915503 | 0.892610495 | 0.938166679 |
| | C(Ethnicity)[T.Black] | -0.792719953 | 0.145208489 | 0.278387872 |
| | C(Ethnicity)[T.Chinese] | 2.910435171 | 0.01170139 | 0.035267445 |
| | C(Ethnicity)[T.Mixed] | 1.468821217 | 0.073211733 | 0.160274334 |
| | C(Ethnicity)[T.Others] | 0.085112122 | 0.894432059 | 0.938166679 |
| Vein Distance tortuosity zone b | C(Age_group)[T.50-59] | -0.064636484 | 0.105633804 | 0.214579012 |
| | C(Age_group)[T.60-69] | -0.177246412 | 8.90E-05 | 0.000437123 |
| | C(Age_group)[T.70+] | -0.290739612 | 0.202452543 | 0.360410021 |
| | C(Sex)[T.Male] | 0.058565105 | 0.087180042 | 0.18341775 |
| | C(Ethnicity)[T.Asian] | 0.259762751 | 0.011980035 | 0.03578052 |
| | C(Ethnicity)[T.Black] | 0.248018869 | 0.022406114 | 0.059020984 |
| | C(Ethnicity)[T.Chinese] | 0.057227175 | 0.803783937 | 0.890663021 |
| | C(Ethnicity)[T.Mixed] | -0.084457828 | 0.605684599 | 0.763586809 |
| | C(Ethnicity)[T.Others] | 0.0571855 | 0.655051882 | 0.806802656 |
| Vein Squared curvature tortuosity zone b | C(Age_group)[T.50-59] | -0.299979969 | 0.292883596 | 0.479264067 |
| | C(Age_group)[T.60-69] | -0.835089521 | 0.009699036 | 0.030071653 |
| | C(Age_group)[T.70+] | -1.399990602 | 0.389978841 | 0.580933998 |
| | C(Sex)[T.Male] | 0.439670932 | 0.072134002 | 0.158450282 |
| | C(Ethnicity)[T.Asian] | 1.420935377 | 0.054188425 | 0.123641195 |

| Feature | Index | Beta | p value | FDR p |
|---|---|---|---|---|
| | C(Ethnicity)[T.Black] | 2.142202633 | 0.005743745 | 0.0192847 |
| | C(Ethnicity)[T.Chinese] | 0.139351641 | 0.93247088 | 0.962699304 |
| | C(Ethnicity)[T.Mixed] | -0.831795042 | 0.476402185 | 0.659633795 |
| | C(Ethnicity)[T.Others] | -0.820746802 | 0.369162405 | 0.559902136 |
| Vein Tortuosity density zone b | C(Age_group)[T.50-59] | 0.000199208 | 0.926480572 | 0.959040592 |
| | C(Age_group)[T.60-69] | -0.004270283 | 0.08054791 | 0.172831277 |
| | C(Age_group)[T.70+] | -0.024867306 | 0.043725359 | 0.103940363 |
| | C(Sex)[T.Male] | 0.002756117 | 0.136414689 | 0.267868843 |
| | C(Ethnicity)[T.Asian] | 0.003335704 | 0.550437284 | 0.722031094 |
| | C(Ethnicity)[T.Black] | -0.000812971 | 0.889843875 | 0.938166679 |
| | C(Ethnicity)[T.Chinese] | 0.030869543 | 0.013179428 | 0.038469682 |
| | C(Ethnicity)[T.Mixed] | -0.013124777 | 0.137782864 | 0.268739185 |
| | C(Ethnicity)[T.Others] | -0.005432375 | 0.432361881 | 0.620881864 |
| CRVE Hubbard zone b | C(Age_group)[T.50-59] | -2.430137583 | 0.000765373 | 0.003061493 |
| | C(Age_group)[T.60-69] | -6.910298181 | 4.87E-17 | 7.51E-16 |
| | C(Age_group)[T.70+] | -17.58658324 | 1.70E-05 | 8.95E-05 |
| | C(Sex)[T.Male] | 1.864930863 | 0.002663529 | 0.009696442 |
| | C(Ethnicity)[T.Asian] | 1.862792187 | 0.317019981 | 0.506676065 |
| | C(Ethnicity)[T.Black] | 1.927347212 | 0.326204618 | 0.511817416 |
| | C(Ethnicity)[T.Chinese] | 7.739755655 | 0.060688708 | 0.135607873 |
| | C(Ethnicity)[T.Mixed] | 2.31636507 | 0.43952344 | 0.627337421 |
| | C(Ethnicity)[T.Others] | 2.984255269 | 0.195061379 | 0.349170645 |
| CRVE Knudtson zone b | C(Age_group)[T.50-59] | -2.741560696 | 0.000472022 | 0.002012305 |
| | C(Age_group)[T.60-69] | -7.658240143 | 1.11E-17 | 1.75E-16 |
| | C(Age_group)[T.70+] | -19.18649122 | 1.55E-05 | 8.22E-05 |
| | C(Sex)[T.Male] | 2.010167353 | 0.00286165 | 0.010301942 |
| | C(Ethnicity)[T.Asian] | 1.885803806 | 0.3508591 | 0.536911715 |
| | C(Ethnicity)[T.Black] | 1.815858641 | 0.39426268 | 0.584615665 |
| | C(Ethnicity)[T.Chinese] | 8.441855818 | 0.059531592 | 0.134412793 |
| | C(Ethnicity)[T.Mixed] | 2.918025477 | 0.369811905 | 0.559902136 |
| | C(Ethnicity)[T.Others] | 3.284804017 | 0.188998987 | 0.3392558 |
| AVR Hubbard zone b | C(Age_group)[T.50-59] | -0.009061978 | 0.949646808 | 0.975231587 |

| Feature | Index | Beta | p value | FDR p |
|---|---|---|---|---|
| | C(Age_group)[T.60-69] | 0.547012438 | 0.000760882 | 0.003061493 |
| | C(Age_group)[T.70+] | 0.131615572 | 0.872400307 | 0.931326852 |
| | C(Sex)[T.Male] | 0.023519251 | 0.848358089 | 0.914723684 |
| | C(Ethnicity)[T.Asian] | -0.880100874 | 0.017804937 | 0.048681853 |
| | C(Ethnicity)[T.Black] | -0.122287744 | 0.753953232 | 0.865486009 |
| | C(Ethnicity)[T.Chinese] | -0.851758652 | 0.303372808 | 0.492695688 |
| | C(Ethnicity)[T.Mixed] | 0.11623867 | 0.843232553 | 0.913736947 |
| | C(Ethnicity)[T.Others] | -0.566675949 | 0.217890829 | 0.38367733 |
| AVR Knudtson zone b | C(Age_group)[T.50-59] | -0.030736642 | 0.798184234 | 0.890229576 |
| | C(Age_group)[T.60-69] | 0.281751181 | 0.038390962 | 0.094232361 |
| | C(Age_group)[T.70+] | 0.222442999 | 0.745905482 | 0.861075312 |
| | C(Sex)[T.Male] | 0.057305569 | 0.578095255 | 0.74032752 |
| | C(Ethnicity)[T.Asian] | -0.856032658 | 0.005937179 | 0.019831401 |
| | C(Ethnicity)[T.Black] | -0.002549505 | 0.993775974 | 0.996099388 |
| | C(Ethnicity)[T.Chinese] | -0.408675525 | 0.555506726 | 0.722065274 |
| | C(Ethnicity)[T.Mixed] | 0.074333934 | 0.879998168 | 0.933287746 |
| | C(Ethnicity)[T.Others] | -0.233015576 | 0.545271909 | 0.719363463 |
| Fractal dimension zone c | C(Age_group)[T.50-59] | -0.012496192 | 2.71E-10 | 2.31E-09 |
| | C(Age_group)[T.60-69] | -0.037349245 | 2.61E-61 | 1.30E-59 |
| | C(Age_group)[T.70+] | -0.052832788 | 2.89E-06 | 1.73E-05 |
| | C(Sex)[T.Male] | 0.0028891 | 0.08804287 | 0.18463359 |
| | C(Ethnicity)[T.Asian] | -0.001273714 | 0.803252287 | 0.890663021 |
| | C(Ethnicity)[T.Black] | -0.004246115 | 0.429275135 | 0.619532935 |
| | C(Ethnicity)[T.Chinese] | 0.02952646 | 0.009575807 | 0.029865606 |
| | C(Ethnicity)[T.Mixed] | 0.001089371 | 0.892917635 | 0.938166679 |
| | C(Ethnicity)[T.Others] | 0.001635972 | 0.796115227 | 0.890229576 |
| Vessel density zone c | C(Age_group)[T.50-59] | -0.000671854 | 2.38E-18 | 4.06E-17 |
| | C(Age_group)[T.60-69] | -0.001690634 | 3.74E-82 | 3.46E-80 |
| | C(Age_group)[T.70+] | -0.002808143 | 1.51E-10 | 1.32E-09 |
| | C(Sex)[T.Male] | 0.000165139 | 0.011982057 | 0.03578052 |
| | C(Ethnicity)[T.Asian] | 0.000658038 | 0.000913304 | 0.003608663 |
| | C(Ethnicity)[T.Black] | 0.000612785 | 0.003291383 | 0.011591393 |

| Feature | Index | Beta | p value | FDR p |
|---|---|---|---|---|
| | C(Ethnicity)[T.Chinese] | 0.000899258 | 0.041963683 | 0.100712839 |
| | C(Ethnicity)[T.Mixed] | 0.000582438 | 0.063635859 | 0.141219303 |
| | C(Ethnicity)[T.Others] | 0.000601263 | 0.014408882 | 0.041256826 |
| Average width zone c | C(Age_group)[T.50-59] | -0.049925848 | 0.690505458 | 0.83479018 |
| | C(Age_group)[T.60-69] | 0.026623571 | 0.851182382 | 0.914723684 |
| | C(Age_group)[T.70+] | 0.040003348 | 0.955447758 | 0.9780887 |
| | C(Sex)[T.Male] | -0.721561158 | 2.06E-11 | 1.99E-10 |
| | C(Ethnicity)[T.Asian] | -0.592275517 | 0.06797258 | 0.150328436 |
| | C(Ethnicity)[T.Black] | -0.730256764 | 0.032221556 | 0.080928558 |
| | C(Ethnicity)[T.Chinese] | 1.846476665 | 0.010682645 | 0.032652612 |
| | C(Ethnicity)[T.Mixed] | 0.30909481 | 0.547293499 | 0.719363463 |
| | C(Ethnicity)[T.Others] | -0.46214067 | 0.250140527 | 0.421015744 |
| Distance tortuosity zone c | C(Age_group)[T.50-59] | 0.01673727 | 0.632930932 | 0.791774602 |
| | C(Age_group)[T.60-69] | 0.056162476 | 0.156792563 | 0.296214521 |
| | C(Age_group)[T.70+] | -0.056118164 | 0.779157291 | 0.88114123 |
| | C(Sex)[T.Male] | -0.150184756 | 5.89E-07 | 3.73E-06 |
| | C(Ethnicity)[T.Asian] | 0.070171411 | 0.439041839 | 0.627337421 |
| | C(Ethnicity)[T.Black] | 0.05747872 | 0.546342081 | 0.719363463 |
| | C(Ethnicity)[T.Chinese] | 0.197603952 | 0.328197324 | 0.513700159 |
| | C(Ethnicity)[T.Mixed] | -0.210691874 | 0.142187165 | 0.274218104 |
| | C(Ethnicity)[T.Others] | 0.030359295 | 0.786911309 | 0.888359805 |
| Squared curvature tortuosity zone c | C(Age_group)[T.50-59] | 0.053520167 | 0.923674945 | 0.957666183 |
| | C(Age_group)[T.60-69] | 1.887569434 | 0.002840734 | 0.010283776 |
| | C(Age_group)[T.70+] | 0.268254475 | 0.932986363 | 0.962699304 |
| | C(Sex)[T.Male] | -2.11681751 | 9.99E-06 | 5.53E-05 |
| | C(Ethnicity)[T.Asian] | 0.223728881 | 0.877001059 | 0.931633912 |
| | C(Ethnicity)[T.Black] | 0.680609591 | 0.654079909 | 0.806802656 |
| | C(Ethnicity)[T.Chinese] | -0.426268954 | 0.894733037 | 0.938166679 |
| | C(Ethnicity)[T.Mixed] | -3.137603807 | 0.170337229 | 0.316270843 |
| | C(Ethnicity)[T.Others] | 0.676594355 | 0.705487098 | 0.844511686 |
| Tortuosity density zone c | C(Age_group)[T.50-59] | -0.001076325 | 0.416623592 | 0.606678848 |
| | C(Age_group)[T.60-69] | -0.006565387 | 1.22E-05 | 6.62E-05 |

| Feature | Index | Beta | p value | FDR p |
|---|---|---|---|---|
| | C(Age_group)[T.70+] | -0.01010667 | 0.181684695 | 0.329953417 |
| | C(Sex)[T.Male] | -0.000512935 | 0.651535293 | 0.806802656 |
| | C(Ethnicity)[T.Asian] | -0.008411263 | 0.014181154 | 0.040841724 |
| | C(Ethnicity)[T.Black] | 0.000529398 | 0.883171154 | 0.935122398 |
| | C(Ethnicity)[T.Chinese] | 0.001895638 | 0.804070783 | 0.890663021 |
| | C(Ethnicity)[T.Mixed] | -0.005419748 | 0.318002334 | 0.506676065 |
| | C(Ethnicity)[T.Others] | -0.008718401 | 0.040090968 | 0.097257526 |
| Artery Fractal dimension zone c | C(Age_group)[T.50-59] | -0.010725616 | 4.26E-10 | 3.54E-09 |
| | C(Age_group)[T.60-69] | -0.030969925 | 3.29E-56 | 1.42E-54 |
| | C(Age_group)[T.70+] | -0.047310925 | 1.39E-06 | 8.43E-06 |
| | C(Sex)[T.Male] | 0.001537826 | 0.295517272 | 0.482355648 |
| | C(Ethnicity)[T.Asian] | -0.005939819 | 0.180773925 | 0.329953417 |
| | C(Ethnicity)[T.Black] | -0.01045372 | 0.024996461 | 0.064790828 |
| | C(Ethnicity)[T.Chinese] | 0.023968223 | 0.015397698 | 0.043761878 |
| | C(Ethnicity)[T.Mixed] | -0.00096406 | 0.890841398 | 0.938166679 |
| | C(Ethnicity)[T.Others] | 0.002069182 | 0.706561259 | 0.844511686 |
| Artery Vessel density zone c | C(Age_group)[T.50-59] | -0.000386701 | 5.18E-25 | 1.08E-23 |
| | C(Age_group)[T.60-69] | -0.000865328 | 1.56E-90 | 2.52E-88 |
| | C(Age_group)[T.70+] | -0.001457128 | 8.36E-12 | 8.46E-11 |
| | C(Sex)[T.Male] | 1.94E-05 | 0.544756948 | 0.719363463 |
| | C(Ethnicity)[T.Asian] | 0.000241713 | 0.012235153 | 0.036202644 |
| | C(Ethnicity)[T.Black] | 9.22E-05 | 0.362799619 | 0.551864208 |
| | C(Ethnicity)[T.Chinese] | 0.000334398 | 0.11984205 | 0.237485164 |
| | C(Ethnicity)[T.Mixed] | 0.000241119 | 0.114325654 | 0.227248539 |
| | C(Ethnicity)[T.Others] | 0.000257912 | 0.030883733 | 0.078174448 |
| Artery Average width zone c | C(Age_group)[T.50-59] | -0.895599294 | 2.50E-11 | 2.31E-10 |
| | C(Age_group)[T.60-69] | -1.158052866 | 2.53E-14 | 3.04E-13 |
| | C(Age_group)[T.70+] | -1.018228721 | 0.183228944 | 0.330730795 |
| | C(Sex)[T.Male] | -0.942707868 | 2.66E-16 | 3.92E-15 |
| | C(Ethnicity)[T.Asian] | 0.345906799 | 0.318370573 | 0.506676065 |
| | C(Ethnicity)[T.Black] | 0.248660354 | 0.494809783 | 0.681191949 |
| | C(Ethnicity)[T.Chinese] | 1.596942177 | 0.038758904 | 0.09477649 |

| Feature | Index | Beta | p value | FDR p |
|---|---|---|---|---|
| | C(Ethnicity)[T.Mixed] | 0.221150166 | 0.686930464 | 0.83357854 |
| | C(Ethnicity)[T.Others] | 0.099539181 | 0.816654228 | 0.895417834 |
| Artery Distance tortuosity zone c | C(Age_group)[T.50-59] | 0.047262706 | 0.514078316 | 0.697534801 |
| | C(Age_group)[T.60-69] | -0.021367057 | 0.79436204 | 0.890229576 |
| | C(Age_group)[T.70+] | -0.121079661 | 0.7697356 | 0.873535321 |
| | C(Sex)[T.Male] | -0.240708496 | 0.000106736 | 0.000512335 |
| | C(Ethnicity)[T.Asian] | -0.180927292 | 0.33440314 | 0.5208972 |
| | C(Ethnicity)[T.Black] | 0.238645381 | 0.22561635 | 0.391955482 |
| | C(Ethnicity)[T.Chinese] | 0.153622187 | 0.713041145 | 0.846246633 |
| | C(Ethnicity)[T.Mixed] | -0.187285201 | 0.527875577 | 0.71244611 |
| | C(Ethnicity)[T.Others] | 0.173019717 | 0.456059993 | 0.642449729 |
| Artery Squared curvature tortuosity zone c | C(Age_group)[T.50-59] | 1.318351191 | 0.349016136 | 0.536911715 |
| | C(Age_group)[T.60-69] | 1.313717313 | 0.409616271 | 0.600681759 |
| | C(Age_group)[T.70+] | 10.29738143 | 0.200240463 | 0.35745405 |
| | C(Sex)[T.Male] | -5.009256497 | 3.34E-05 | 0.000170631 |
| | C(Ethnicity)[T.Asian] | -5.162248796 | 0.156461193 | 0.296214521 |
| | C(Ethnicity)[T.Black] | 3.888696464 | 0.30963813 | 0.500362864 |
| | C(Ethnicity)[T.Chinese] | -7.421144478 | 0.360647758 | 0.549881758 |
| | C(Ethnicity)[T.Mixed] | -5.449988962 | 0.344573744 | 0.532896864 |
| | C(Ethnicity)[T.Others] | 3.679086697 | 0.414785185 | 0.605955894 |
| Artery Tortuosity density zone c | C(Age_group)[T.50-59] | -0.000718343 | 0.679940478 | 0.828198176 |
| | C(Age_group)[T.60-69] | -0.008789859 | 8.32E-06 | 4.73E-05 |
| | C(Age_group)[T.70+] | 0.010263862 | 0.301995842 | 0.491691722 |
| | C(Sex)[T.Male] | -0.001766048 | 0.236723734 | 0.407970691 |
| | C(Ethnicity)[T.Asian] | -0.012761241 | 0.00463534 | 0.015808948 |
| | C(Ethnicity)[T.Black] | -0.005475825 | 0.247443364 | 0.418650914 |
| | C(Ethnicity)[T.Chinese] | -0.015977094 | 0.111621082 | 0.224628761 |
| | C(Ethnicity)[T.Mixed] | -0.01187675 | 0.09590233 | 0.196660474 |
| | C(Ethnicity)[T.Others] | -0.011976122 | 0.031891558 | 0.080411399 |
| CRAE Hubbard zone c | C(Age_group)[T.50-59] | -4.028173747 | 1.05E-32 | 2.62E-31 |
| | C(Age_group)[T.60-69] | -7.5646505 | 3.23E-85 | 3.48E-83 |
| | C(Age_group)[T.70+] | -11.51492528 | 2.11E-09 | 1.65E-08 |

| Feature | Index | Beta | p value | FDR p |
|---|---|---|---|---|
| | C(Sex)[T.Male] | -0.871242668 | 0.002524248 | 0.009293824 |
| | C(Ethnicity)[T.Asian] | 1.379742674 | 0.112772041 | 0.225544081 |
| | C(Ethnicity)[T.Black] | -0.092910515 | 0.91903842 | 0.957454817 |
| | C(Ethnicity)[T.Chinese] | 4.775083676 | 0.013802832 | 0.039929622 |
| | C(Ethnicity)[T.Mixed] | 2.610123578 | 0.058069678 | 0.131570458 |
| | C(Ethnicity)[T.Others] | 1.082314126 | 0.315133757 | 0.506676065 |
| CRAE Knudtson zone c | C(Age_group)[T.50-59] | -3.703501131 | 1.67E-33 | 4.33E-32 |
| | C(Age_group)[T.60-69] | -6.957907704 | 2.14E-87 | 2.78E-85 |
| | C(Age_group)[T.70+] | -10.68838445 | 9.09E-10 | 7.36E-09 |
| | C(Sex)[T.Male] | -0.754106566 | 0.003970656 | 0.013613678 |
| | C(Ethnicity)[T.Asian] | 1.321576959 | 0.094215663 | 0.194432324 |
| | C(Ethnicity)[T.Black] | -0.058240146 | 0.944034867 | 0.971007292 |
| | C(Ethnicity)[T.Chinese] | 4.246782468 | 0.015829883 | 0.044598976 |
| | C(Ethnicity)[T.Mixed] | 2.47583132 | 0.047638144 | 0.110248276 |
| | C(Ethnicity)[T.Others] | 0.961538172 | 0.325491558 | 0.511817416 |
| Vein Fractal dimension zone c | C(Age_group)[T.50-59] | 0.065369576 | 0.060653359 | 0.135607873 |
| | C(Age_group)[T.60-69] | 0.260986036 | 3.28E-11 | 2.99E-10 |
| | C(Age_group)[T.70+] | 0.352878201 | 0.074874586 | 0.162814535 |
| | C(Sex)[T.Male] | 0.009561416 | 0.748125619 | 0.861075312 |
| | C(Ethnicity)[T.Asian] | 0.162354147 | 0.074358819 | 0.162237423 |
| | C(Ethnicity)[T.Black] | 0.152281837 | 0.111199515 | 0.224477525 |
| | C(Ethnicity)[T.Chinese] | -0.381403433 | 0.060078897 | 0.135177519 |
| | C(Ethnicity)[T.Mixed] | 0.056293678 | 0.695951904 | 0.8392306 |
| | C(Ethnicity)[T.Others] | 0.084661038 | 0.450493372 | 0.636938746 |
| Vein Vessel density zone c | C(Age_group)[T.50-59] | -0.000352237 | 6.34E-13 | 6.74E-12 |
| | C(Age_group)[T.60-69] | -0.000881079 | 1.08E-55 | 4.39E-54 |
| | C(Age_group)[T.70+] | -0.00108089 | 0.00041739 | 0.001791185 |
| | C(Sex)[T.Male] | 0.000169673 | 5.42E-05 | 0.000272297 |
| | C(Ethnicity)[T.Asian] | 0.000853369 | 2.21E-11 | 2.11E-10 |
| | C(Ethnicity)[T.Black] | 0.001060443 | 3.84E-15 | 5.41E-14 |
| | C(Ethnicity)[T.Chinese] | 0.000725821 | 0.009416937 | 0.029622209 |
| | C(Ethnicity)[T.Mixed] | 0.000488875 | 0.018153834 | 0.049427245 |

| Feature | Index | Beta | p value | FDR p |
| --- | --- | --- | --- | --- |
| | C(Ethnicity)[T.Others] | 0.00078766 | 1.16E-06 | 7.09E-06 |
| Vein Average width zone c | C(Age_group)[T.50-59] | 0.006644848 | 0.97176846 | 0.986999941 |
| | C(Age_group)[T.60-69] | 0.923084151 | 1.49E-05 | 7.97E-05 |
| | C(Age_group)[T.70+] | 1.746230291 | 0.137776792 | 0.268739185 |
| | C(Sex)[T.Male] | -0.555695704 | 0.000580068 | 0.002420268 |
| | C(Ethnicity)[T.Asian] | -0.571110101 | 0.243012279 | 0.414167334 |
| | C(Ethnicity)[T.Black] | -0.415543853 | 0.421598276 | 0.609811792 |
| | C(Ethnicity)[T.Chinese] | 3.029307036 | 0.004807053 | 0.016308746 |
| | C(Ethnicity)[T.Mixed] | 0.600291689 | 0.45025141 | 0.636938746 |
| | C(Ethnicity)[T.Others] | -0.200614971 | 0.746999735 | 0.861075312 |
| Vein Distance tortuosity zone c | C(Age_group)[T.50-59] | 0.049216105 | 0.418154705 | 0.607543159 |
| | C(Age_group)[T.60-69] | 0.038740544 | 0.57423887 | 0.739660062 |
| | C(Age_group)[T.70+] | -0.254496202 | 0.50403793 | 0.686672799 |
| | C(Sex)[T.Male] | -0.051687756 | 0.322648918 | 0.50994268 |
| | C(Ethnicity)[T.Asian] | 0.407674928 | 0.010065724 | 0.031059949 |
| | C(Ethnicity)[T.Black] | 0.289358141 | 0.083925939 | 0.178308225 |
| | C(Ethnicity)[T.Chinese] | 0.01470349 | 0.96626857 | 0.985889932 |
| | C(Ethnicity)[T.Mixed] | -0.061229127 | 0.81198146 | 0.894836711 |
| | C(Ethnicity)[T.Others] | 0.169294398 | 0.400431024 | 0.58972569 |
| Vein Squared curvature tortuosity zone c | C(Age_group)[T.50-59] | 0.208263453 | 0.820162952 | 0.896232028 |
| | C(Age_group)[T.60-69] | 1.541174347 | 0.138102081 | 0.268739185 |
| | C(Age_group)[T.70+] | 5.368561671 | 0.349682824 | 0.536911715 |
| | C(Sex)[T.Male] | -0.018399857 | 0.981361109 | 0.992859331 |
| | C(Ethnicity)[T.Asian] | 6.949203317 | 0.003607227 | 0.012499911 |
| | C(Ethnicity)[T.Black] | 5.354601909 | 0.033832814 | 0.084321782 |
| | C(Ethnicity)[T.Chinese] | 1.870911993 | 0.721060477 | 0.851138601 |
| | C(Ethnicity)[T.Mixed] | 1.931236148 | 0.618605644 | 0.776853599 |
| | C(Ethnicity)[T.Others] | 1.696843831 | 0.576004333 | 0.739660062 |
| Vein Tortuosity density zone c | C(Age_group)[T.50-59] | -0.001220812 | 0.531369708 | 0.714051913 |
| | C(Age_group)[T.60-69] | -0.007616908 | 0.000579839 | 0.002420268 |
| | C(Age_group)[T.70+] | -0.031931346 | 0.009002003 | 0.028455112 |
| | C(Sex)[T.Male] | 0.004015554 | 0.016657175 | 0.046525212 |

| Feature | Index | Beta | p value | FDR p |
|---|---|---|---|---|
| | C(Ethnicity)[T.Asian] | 0.006905847 | 0.174149965 | 0.322426221 |
| | C(Ethnicity)[T.Black] | 0.007487223 | 0.163357874 | 0.305322074 |
| | C(Ethnicity)[T.Chinese] | 0.009653747 | 0.38687097 | 0.577632231 |
| | C(Ethnicity)[T.Mixed] | -0.007854284 | 0.34163015 | 0.529608462 |
| | C(Ethnicity)[T.Others] | -0.007775642 | 0.228743158 | 0.395268176 |
| CRVE Hubbard zone c | C(Age_group)[T.50-59] | -3.185556781 | 1.13E-14 | 1.41E-13 |
| | C(Age_group)[T.60-69] | -6.73227676 | 2.48E-46 | 6.99E-45 |
| | C(Age_group)[T.70+] | -10.05619578 | 9.60E-05 | 0.000467828 |
| | C(Sex)[T.Male] | 2.258853048 | 1.83E-10 | 1.58E-09 |
| | C(Ethnicity)[T.Asian] | 2.867588883 | 0.007449116 | 0.024086658 |
| | C(Ethnicity)[T.Black] | 5.96036176 | 1.61E-07 | 1.06E-06 |
| | C(Ethnicity)[T.Chinese] | 8.093839136 | 0.000582657 | 0.002420268 |
| | C(Ethnicity)[T.Mixed] | 2.313525203 | 0.183974879 | 0.331154782 |
| | C(Ethnicity)[T.Others] | 3.801857594 | 0.005259937 | 0.017752287 |
| CRVE Knudtson zone c | C(Age_group)[T.50-59] | -3.717328045 | 1.08E-14 | 1.37E-13 |
| | C(Age_group)[T.60-69] | -7.894460039 | 7.83E-47 | 2.31E-45 |
| | C(Age_group)[T.70+] | -11.58079854 | 0.000116941 | 0.000545165 |
| | C(Sex)[T.Male] | 2.672706967 | 9.87E-11 | 8.89E-10 |
| | C(Ethnicity)[T.Asian] | 3.342279192 | 0.007471324 | 0.024086658 |
| | C(Ethnicity)[T.Black] | 7.003424839 | 1.29E-07 | 8.63E-07 |
| | C(Ethnicity)[T.Chinese] | 8.814831896 | 0.001313782 | 0.005037461 |
| | C(Ethnicity)[T.Mixed] | 2.721696233 | 0.180073462 | 0.329953417 |
| | C(Ethnicity)[T.Others] | 4.350584565 | 0.006164335 | 0.020380047 |
| AVR Hubbard zone c | C(Age_group)[T.50-59] | -0.028043363 | 0.528837313 | 0.71244611 |
| | C(Age_group)[T.60-69] | -0.002604601 | 0.958875217 | 0.980049117 |
| | C(Age_group)[T.70+] | -0.063919589 | 0.818796712 | 0.896232028 |
| | C(Sex)[T.Male] | -0.031191861 | 0.415192002 | 0.605955894 |
| | C(Ethnicity)[T.Asian] | -0.030759789 | 0.790885292 | 0.889745954 |
| | C(Ethnicity)[T.Black] | 0.202983719 | 0.097892981 | 0.200109311 |
| | C(Ethnicity)[T.Chinese] | -0.030649565 | 0.904214893 | 0.945020651 |
| | C(Ethnicity)[T.Mixed] | -0.036224571 | 0.847651288 | 0.914723684 |
| | C(Ethnicity)[T.Others] | -0.044943497 | 0.760562259 | 0.86921401 |

| Feature | Index | Beta | p value | FDR p |
|---|---|---|---|---|
| AVR Knudtson zone c | C(Age_group)[T.50-59] | -0.02014572 | 0.540206931 | 0.717323958 |
| | C(Age_group)[T.60-69] | -0.003586684 | 0.923415279 | 0.957666183 |
| | C(Age_group)[T.70+] | -0.048636314 | 0.813437253 | 0.894919083 |
| | C(Sex)[T.Male] | -0.024515332 | 0.385958697 | 0.577601007 |
| | C(Ethnicity)[T.Asian] | -0.02273045 | 0.790797061 | 0.889745954 |
| | C(Ethnicity)[T.Black] | 0.12794391 | 0.15781599 | 0.297281283 |
| | C(Ethnicity)[T.Chinese] | -0.022299656 | 0.90564479 | 0.945020651 |
| | C(Ethnicity)[T.Mixed] | -0.026126248 | 0.851201206 | 0.914723684 |
| | C(Ethnicity)[T.Others] | -0.034046991 | 0.754629005 | 0.865486009 |

All ethnicity is with respect to White, Age group with respect to 40-49 years, and Sex with relation to female
